# Design, Synthesis, Anticancer Activity and Molecular Docking of New 1,2,3-Triazole-Based Glycosides Bearing 1,3,4-Thiadiazolyl, Indolyl and Arylacetamide Scaffolds

**DOI:** 10.3390/molecules27206960

**Published:** 2022-10-17

**Authors:** Hussein H. Elganzory, Fahad M. Alminderej, Mohamed N. El-Bayaa, Hanem M. Awad, Eman S. Nossier, Wael A. El-Sayed

**Affiliations:** 1 Department of Chemistry, College of Science, Qassim University, Buraidah 51452, Saudi Arabia; 2 Photochemistry Department, National Research Centre, Cairo 12622, Egypt; 3 Tanning Materials and Leather Technology Department, National Research Centre, El-Behouth St, Dokki, Cairo 12622, Egypt; 4 Department of Pharmaceutical Medicinal Chemistry and Drug Design, Faculty of Pharmacy (Girls), Al-Azhar University, Cairo 11754, Egypt

**Keywords:** triazole, indole, glycosides, thiadiazole, arylacetamide, EGFR, HCT-116, MCF-7, anticancer

## Abstract

New 1,3,4-thiadiazole thioglycosides linked to a substituted arylidine system were synthesized via heterocyclization via click 1,3-dipolar cycloaddition. The click strategy was used for the synthesis of new 1,3,4-thiadiazole and 1,2,3-triazole hybrid glycoside-based indolyl systems as novel hybrid molecules by reacting azide derivatives with the corresponding acetylated glycosyl terminal acetylenes. The cytotoxic activities of the compounds were studied against HCT-116 (human colorectal carcinoma) and MCF-7 (human breast adenocarcinoma) cell lines using the MTT assay. The results showed that the key thiadiazolethione compounds, the triazole glycosides linked to p-methoxyarylidine derivatives and the free hydroxyl glycoside had potent activity comparable to the reference drug, doxorubicin, against MCF-7 human cancer cells. Docking simulation studies were performed to check the binding patterns of the synthesized compounds. Enzyme inhibition assay studies were also conducted for the epidermal growth factor receptor (EGFR), and the results explained the activity of a number of derivatives.

## 1. Introduction

The heightened global burden of cancer in recent decades has driven extensive research to discover effective and selective anticancer candidates. Because of the resistance and severe toxicity associated with current drugs, there is a great demand to provide more efficient therapeutic strategies and molecular targets [1,2].

**Figure 1:**
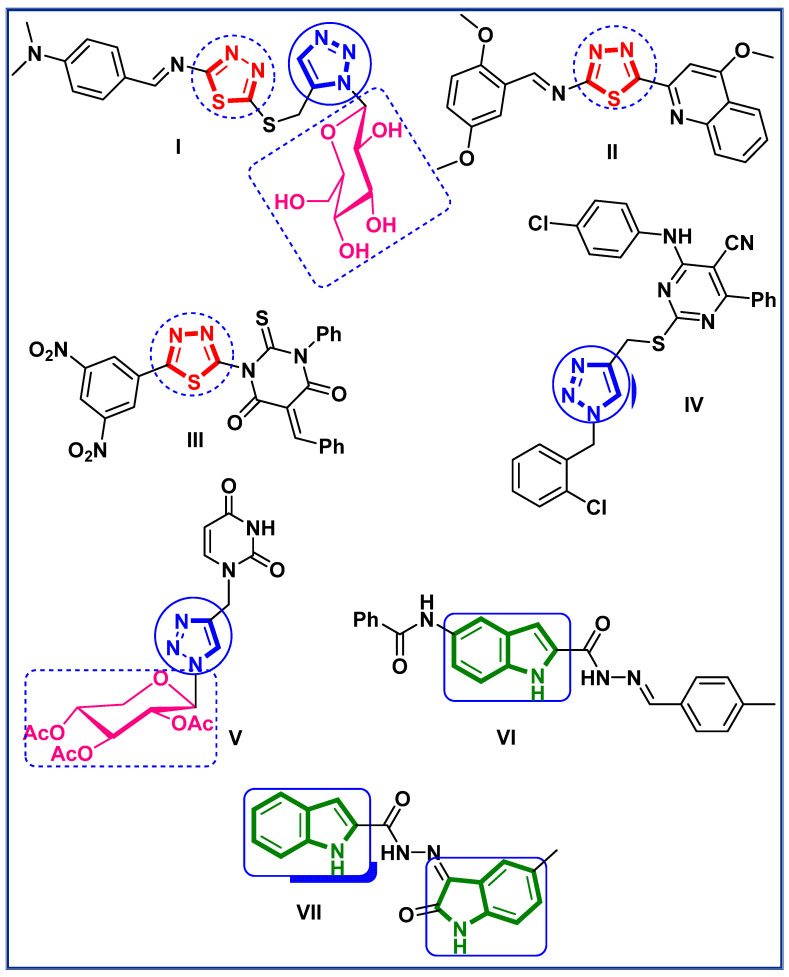
Reported anticancer agents bearing 1,3,4-thiadiazol, 1,2,3-triazole, indole and glycoside fragments.

Heterocyclic moieties bearing nitrogen atoms, such as 1,3,4-thiadiazole and 1,2,3-triazole, play a critical role in medicinal chemistry and are associated with different pharmaceutical properties, such as anticancer, antimicrobial, antidiabetic, anti-inflammatory and antihypertensive [3,4,5,6,7,8,9,10,11,12,13,14,15] effects. The reported targets (**I**–**V**, Figure 1) with these scaffolds revealed potent cytotoxic activities against various human cancer cell lines with different mechanisms, such as the inhibition of the dihydrofolate reductase enzyme and NUDT5 (Nudix Hydrolase 5) to silence hormone signaling in breast cancer [16,17,18,19].

As a privileged scaffold, indole displays a promising and leading heterocyclic nucleus that provides it with a range of attractive biological activities, chiefly antitumor ones [20]. The indole derivatives **VI** and **VII** (Figure 1) were found to exert their anticancer activities through the inhibition of cyclin-dependent kinases CDK-9 and CDK-4, respectively [21,22].

Recently, several pharmacophores containing glycoside fragments, e.g., **I** and **V** (Figure 1), have been developed and reported to have potential antitumor activity [23,24,25].

**Figure 2:**
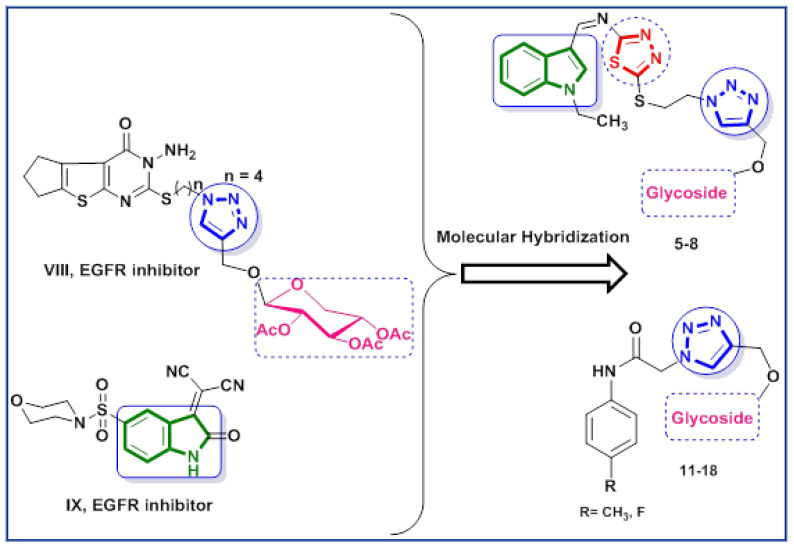
Reported EGFR inhibitors and the design concept of glycosides linked to indole–thiadiazole–triazole bases or substituted arylacetamino–triazole hybrids.

The epidermal growth factor receptor (EGFR) belongs to a fundamental subfamily of protein kinases that contains four members: EGFR (HER-1), HER-2, HER-3 and HER-4. EGFR is a crucial mediator performing a very vital role in the regulation of essential cellular processes involving proliferation, migration and survival [26]. The overexpression of EGFR is one of the most widely investigated for its role in the progression of many human solid tumors, such as breast cancer, non-small cell lung cancer and hepatocellular carcinoma [27]. In addition, the amplification of the HER-2 oncogene is detected in 15–30% of women suffering from breast cancer [28], which is regarded as the most serious subtype [29,30]. Attributable to the essential role of overactive EGFR and HER-2 tyrosine kinases, the inhibition of these enzymes is a well-known effective target for anticancer therapies [31,32]. The most representative drugs in this class are afatinib, lapatinib, gefitinib and erlotinib, which have been used clinically for targeted anticancer therapies [33]. Lately, it has been observed that the triazole glycoside **VIII** and the indole analog **IX** (Figure 2) demonstrate strong cytotoxic potency against different cancer cells through their EGFR inhibitory activity [19,34].

Molecular hybridization is an effective and simple tool to covalently merge two drug pharmacophores to obtain a single molecule [35]. Considering the facts discussed above regarding the biological significance of indole, thiadiazole, triazole and glycoside fragments (Figure 2) and upon the continuation of our research in the discovery of potent anticancer targets [36,37,38,39,40,41], it was conceived that the joining of these pharmacophores into one nucleus through a fragment-based drug design approach would develop highly potent anticancer targets. Herein, a novel series of glycosides incorporated into indole–thiadiazole–triazole bases or substituted arylacetamino–triazole hybrids were designed, synthesized and evaluated for their cytotoxic activities. A kinase inhibition assessment against EGFR^WT^, EGFR^T790M^ and HER-2 was performed for the promising analogs to detect their mechanism of action. Furthermore, the apoptotic activity of the most potent derivatives was estimated by cell cycle analysis. Molecular docking at the binding sites of EGFR^WT^, EGFR^T790M^ and HER-2 receptors was carried out in order to gain insight into new anticancer leads.

## 2. Results and Discussion

### 2.1. Chemistry

The synthetic strategy for the preparation of the targeted glycosides involved two different pathways to obtain two types of hybrid compounds; the first represents new glycosides of indole–thiadiazole–triazole hybrid bases, and the other is a group of substituted arylacetamino–triazole glycosides. The starting 1,3,4-thiadiazolylindole derivative **1** was prepared as previously reported [42]. The alkylation of the functionalized 1,3,4-thiadiazolylindole compound **1** with 1,2-dibromoethane afforded the alkylated derivative **2**, which contains an *S*-substituted bromoethyl side chain, followed by azidation with sodium azide, leading to the formation of the corresponding functionalized azide derivative **3**. The infrared spectra of the azide product **3** displayed the distinctive bands of the azide group, and its corresponding ^1^H NMR spectrum revealed the triplet signals of methylene groups in the side-chain substituent attached to the thiadiazole ring.

**Scheme 1:**
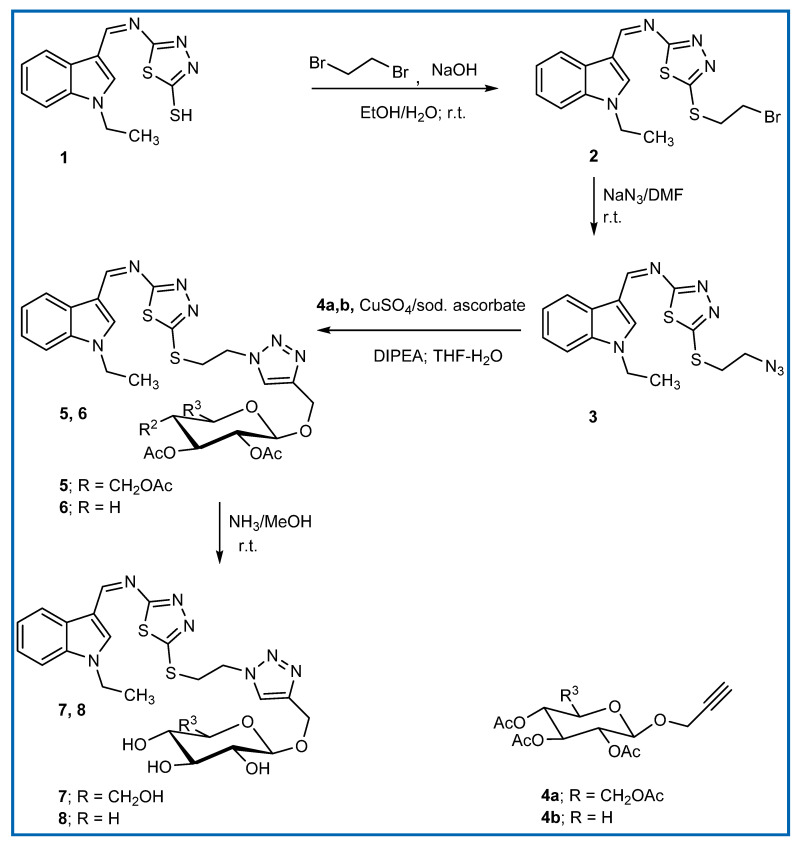
Synthesis of indolyl-thiadiazolyl-1,2,3-triazolyl hybrid glycosides.

The synthesized azide **3** was reacted with the propargyl glycosides of the gluco- and xylopyranosyl moieties **4a,b** under click conditions by a Cu-catalyzed cycloaddition reaction (CuCAAC) and resulted in the formation of the 1,2,3-triazole glycosides **5** and **6**, respectively. Sodium ascorbate and copper sulfate were used for the generation of the required Cu(I) catalyst by the in situ reduction of Cu(II) in the reaction medium. Upon testing a number of solvent systems, the THF–H_2_O mixture (2:1) was determined to be the best medium and resulted in the formation of the targeted 1,2,3-triazole products. The other tested media systems in the click reaction were a *^t^*BuOH/H_2_O mixture (2:1) and a mixture of H_2_O/*^t^*BuOH/CH_2_Cl_2_ (1:2:8), which, after 2 days and 55 h with stirring at 60 °C, respectively, had yields lower than 52%. In the structures of the latter glycosides, the glycosyl moiety was attached to C^4^ of the triazole system as analogs of modified *C*-nucleosides. The IR spectra of compounds **5** and **6** demonstrated characteristic bands related to the carbonyl frequency in the acetate group at their known assigned values. Their ^1^H NMR spectra exhibited signals attributed to the sugar fragments, which were assigned to the formed *β*-confirmation of the triazole–sugar linkage, as indicated by the coupling constant of the anomeric proton. A deprotection reaction of the acetylated glycosides **5** and **6** was achieved under the action of a saturated ammonia solution in methanol and afforded the triazole glycosides **7** and **8** with free hydroxyl groups, which was in accord with their spectral data (Scheme 1).

**Scheme 2:**
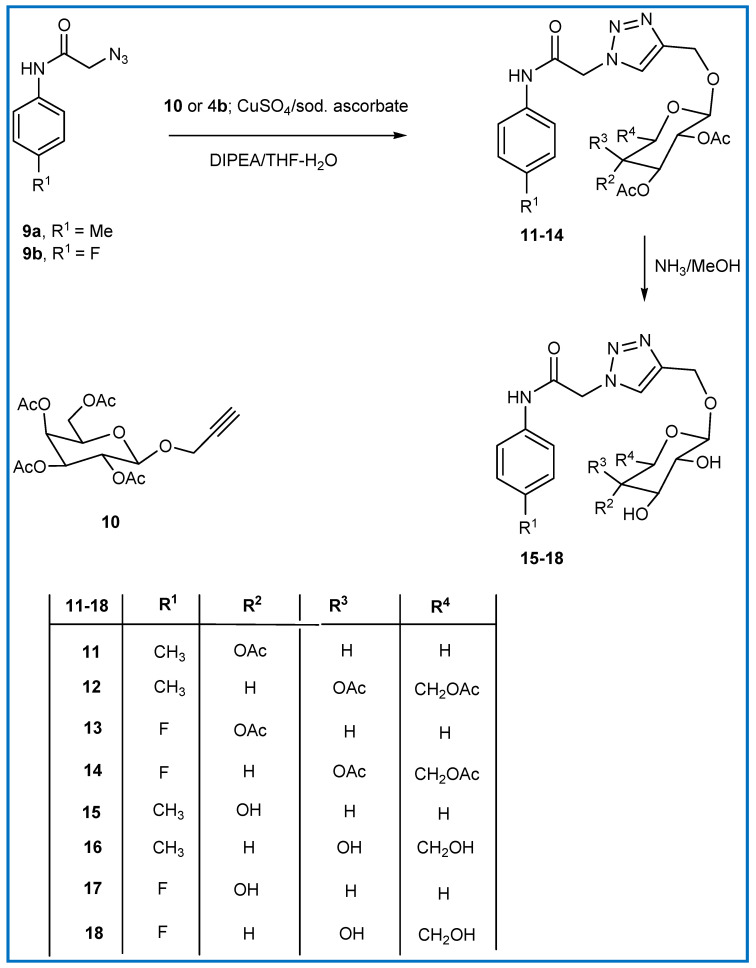
Synthesis of arylaminoacetamide triazolyl glycosides.

The click synthetic approach was also applied for two other functionalized azide compounds, **9a,b**, incorporating a substituted aryl acetamide system. Accordingly, in this way, the reaction was performed to realize the linkage of the terminal acetylenic sugar with **9a,b** to obtain the 1,2,3-triazole glycosides **11**–**14** (Scheme 2). 

^1^H NMR of the formed substituted glycosyl heterocycles showed all of the hydrogens in the formed compounds at their characteristic regions, revealing the β-configuration of the attachment of the sugar part. The latter triazolyl-glycosides **11**–**14** were hydrolyzed to afford the deacetylated derivatives **15**–**18**, respectively, by using a methanolic ammonia solution (Scheme 2). The absorption bands characterized the hydroxyl groups of the afforded deacetylated glycosidic compounds at their characteristic regions, in addition to the disappearance of the acetyl-carbonyl absorption bands. Furthermore, the NMR spectra indicated the deacetylation process and revealed the existence of hydroxyl proton signals and the disappearance of the signals of the methyl protons of the acetyl groups. The purity of the compounds was revealed by the adequate results of the applied spectral and analytical analyses, such as ^1^H and ^13^C NMR, IR and elemental analysis, for the structural confirmation of the synthesized compounds, in addition to the TLC technique, and the proportions of the afforded products indicated a sufficient degree of purity.

### 2.2. Biological Evaluation

#### 2.2.1. Cytotoxic Screening

The synthesized compounds **1**–**3**, **5**–**9** and **11**–**18** were all examined in vitro for their cytotoxic activities against HCT-116 and MCF-7 human cancer cells using the MTT assay, and doxorubicin was applied as a reference drug. All compounds suppressed both cancer cells in a dose-dependent manner, and the results are depicted in Table 1 and Figures S1–S3 (Supplementary Data). In the case of HCT-116 human colorectal carcinoma cells, compounds **12**, **8** and **15** had more potent cytotoxic activities (IC_50_ = 2.2, 4.6 and 11.4 µM, respectively); both compounds **11** and **7** had comparable and promising cytotoxic activities (IC_50_ = 15.4 and 15.5 µM, respectively), and all other compounds had significantly less cytotoxic activities (IC_50_ range 20.8–52.7 µM) relative to that of doxorubicin (IC_50_ = 12.1 µM). In the case of MCF-7 human breast cancer cells, six compounds (**7**, **15**, **11**, **3**, **8** and **12**) had more potent cytotoxicity (IC_50_ = 0.5, 0.6, 0.8, 1.1, 4.2 and 5.7 µM, respectively); three compounds (**6**, **5** and **2**) showed promising cytotoxic potency (IC_50_ = 10.8, 13.9 and 14.1 µM, respectively), and the rest of the compounds had significantly less cytotoxic activities (IC_50_ range 18.1–35.3 µM) relative to the reference drug (IC_50_ = 9.4 µM).

#### 2.2.2. Structure–Activity Relationship

The incorporation of 1-ethyl-1*H*-indole into 1,3,4-thiadiazol-2-thiol through the methanimine group (CH=N) in compound **1** afforded weak cytotoxic activity against HCT-116 and moderate activity (about half the activity of doxorubicin) against MCF-7 (IC_50_ = 46.9 ± 4.7 and 18.1 ± 2.4 µM against HCT-116 and MCF-7, respectively). The substitution of the thiol group with 2-bromoethyl afforded compound **2**, with a slight improvement in the cytotoxicity against MCF-7 only (IC_50_ = 14.1 ± 3.1 µM), while substitution with the 2-azidoethyl moiety in compound **3** yielded excellent activity against MCF-7 and moderate activity against HCT-116 (IC_50_ = 1.1 ± 0.6 and 30.8 ± 4.3 µM against MCF-7 and HCT-116, respectively). A noticeable improvement in activity was observed against both screened cell lines upon substitution with acetylated 1,2,3-triazole glycosides in **6** and **5** (IC_50_ = 20.8 ± 2.1 and 22.6 ± 2.2 µM against HCT-116; 10.8 ± 1.5 and 13.9 ± 2.1 against MCF-7, respectively). Replacement with deacetylated N-glycosides in **7** and **8** revealed excellent and more potent cytotoxicity against both cancerous cell lines (IC_50_ = 15.5 ± 1.8 and 4.6 ± 0.9 µM against HCT-116; 0.5 ± 0.2 and 4.2 ± 1.1 against MCF-7, respectively).

Two series were obtained upon the fixation of the 1,2,3-triazole glycoside scaffold due to its detectable importance in the enhancement of cytotoxicity and the replacement of the 1,3,4-thiadiazol-2-yl)-1-(1-ethyl-1*H*-indol-3-yl)methanimine fragment in Scheme 1 with (p-fluorophenyl or p-tolyl)acetamide in Scheme 2. It was observed that the starting materials 2-azido-N-(p-fluorophenyl or p-tolyl)acetamide **9b** and **9a** showed weak anticancer activity (IC_50_ = 33.3 ± 3.9 and 42.3 ± 5.8 µM against HCT-116; 26.4 ± 3.1 and 24.1 ± 2.9 against MCF-7, respectively). The series bearing a p-tolylacetamide moiety with an electron-donating group (CH_3_) in **11**, **12** and **15** elevated the potency over the reference against both cancer cell lines, except in **16** (IC_50_ range for **11**, **12** and **15** from 2.2 ± 0.5 to 15.4 ± 2.1 µM against HCT-116 and from 0.6 ± 1.5 to 5.7 ±1.1 against MCF-7, respectively), while the other one bearing p-fluorophenylacetamide with an electron-withdrawing group displayed weak activity against the screened cell lines.

Finally, combined with our previous study [16], it can be concluded that the promising cytotoxic activity is attributed to the existence of the 1,2,3-triazole-deacetylated N-glycoside hybrid. Furthermore, the incorporation of indole and 1,3,4-thiadiazole scaffolds provided an opportunity for more potency, and they could be selected as leads for the discovery of anticancer agents in the future.

#### 2.2.3. In Vitro Inhibitory Assessment of EGFR^WT^, EGFR^T790M^ and HER-2 

Regarding the previous cytotoxic potency, the triazole-based glycosides **7**, **8**, **11**, **12** and **15** were chosen for further elucidation of their EGFR^WT^, EGFR^T790M^ and HER-2 inhibitory activities using erlotinib as a reference drug. As illustrated in Table 2, compounds **7**, **8** and **15** displayed the highest potential inhibitory activities, with IC_50_ ranges of 0.21–0.38, 1.50–1.95 and 2.18–2.77 µM against EGFR^WT^, EGFR^T790M^ and HER-2, respectively, but they were slightly less than that of erlotinib (IC_50_ = 0.13, 0.62 and 1.40 µM). However, the remaining derivatives **11** and **12** exhibited a drastic decrease in the suppression effect (IC_50_ = 6.45 and 12.70 µM against EGFR^WT^; 11.80 and 15.22 µM against EGFR^T790M^; and 8.60 and 18. 40 µM against HER-2, respectively), which might be ascribed to a poor fit within the EGFR^WT^, EGFR^T790M^ and HER-2 binding pockets.

#### 2.2.4. Detection of Apoptosis and Cell Cycle Analysis

The annexin V/PI binding assay was carried out on derivatives **7** and **15**, which showed the most potent cytotoxicity and kinase inhibitory activity, to examine their apoptotic effects on the MCF-7 cancer cells. These cells were stained with two dyes, annexin V/propidium iodide (PI), after treating them with derivatives **7** and **15** at their IC_50_ concentrations of 0.5 and 0.6 μM for 24 h through the flow cytometry method [43]. It was observed that there was an increase in the percentages of late apoptosis caused by the tested compounds **7** and **15** from 0.28% (control DMSO/MCF-7 cells) to 18.81% and 25.48%, respectively. Furthermore, the screened derivatives exhibited early apoptotic effects of 4.39% and 2.69% compared to 0.46% in untreated MCF-7 cells, with necrosis percentages of 11.41% and 14.28%, respectively, versus 1.25% produced by the DMSO control (Table 3) (Figures S4 and S5, Supplementary Data). The proportion of late apoptosis obtained with compounds **7** and **15** was higher than that of the early phase, leading to the induction of apoptosis and confirming the effectiveness of these derivatives in cancer therapy. 

**Figure 3:**
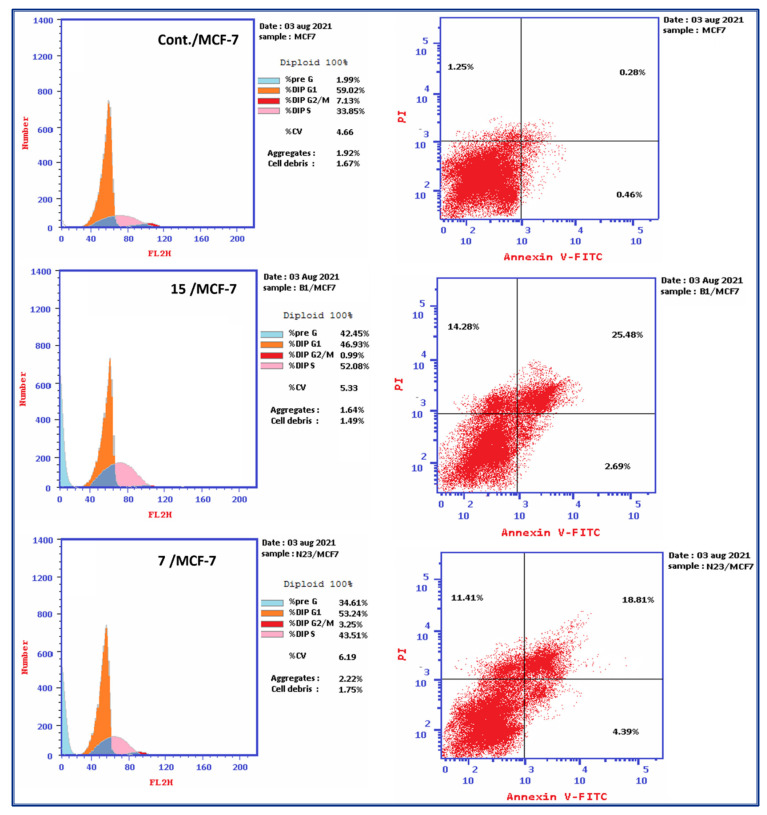
Analysis of the cell cycle and the effect of derivatives **7** and **15** on the percentage of annexin V-FITC-positive staining in MCF-7 cells in comparison with the control.

Moreover, the cessation of cell division at cell cycle checkpoints is considered one of the main roles of anticancer therapeutics [44]. The data presented in Figure 3 and Figure S5 demonstrate that there was cell accumulation at sub-G1 and S phases, with percentages of 34.61% and 43.51% in MCF-7 cells treated with compound **7** and 42.45% and 52.08% in those treated with compound **15**, compared to 1.99% and 33.85% in the untreated MCF-7 cells, respectively. The previous findings clearly show that derivatives **7** and **15** triggered a significant cell cycle arrest at sub-G1 and S phases compared with the untreated MCF-7 cells.

### 2.3. Molecular Docking Study

Docking simulations for targets **7**, **8** and **15** were performed using Molecular Operating Environment (MOE-Dock) software version 2014.0901 [45,46] to understand their binding modes within the EGFR^WT^, EGFR^T790M^ and HER-2 active sites and to interpret their promising in vitro inhibitory activities. The Protein Data Bank files (PDB codes: 1M17, 3UG2 and 3RCD) were chosen and downloaded for the corresponding EGFR^WT^, EGFR^T790M^ and HER-2 enzymes, respectively [46,47,48]. The native ligands erlotinib, gefitinib and TAK-285 complexed with the co-crystallized structures of these enzymes were redocked to validate the docking processes and afforded energy scores of −12.80, −11.64 and −12.33 kcal/mol, with root-mean-square deviation (RMDS) values of 0.88, 0.75 and 1.23 Å, respectively.

As previously reported [47,48,49], erlotinib and gefitinib, bearing quinazoline scaffolds, formed H-bonds between the nitrogen at p-1 and the key amino acids **Met769** and **Met793** within the active sites of EGFR^WT^ and EGFR^T790M^, respectively. In addition, the N-1 of pyrrolo[3,2-*d*]pyrimidine in the ligand TAK-285 displayed hydrogen bonding with **Met801** within the binding site of HER-2.

**Figure 4:**
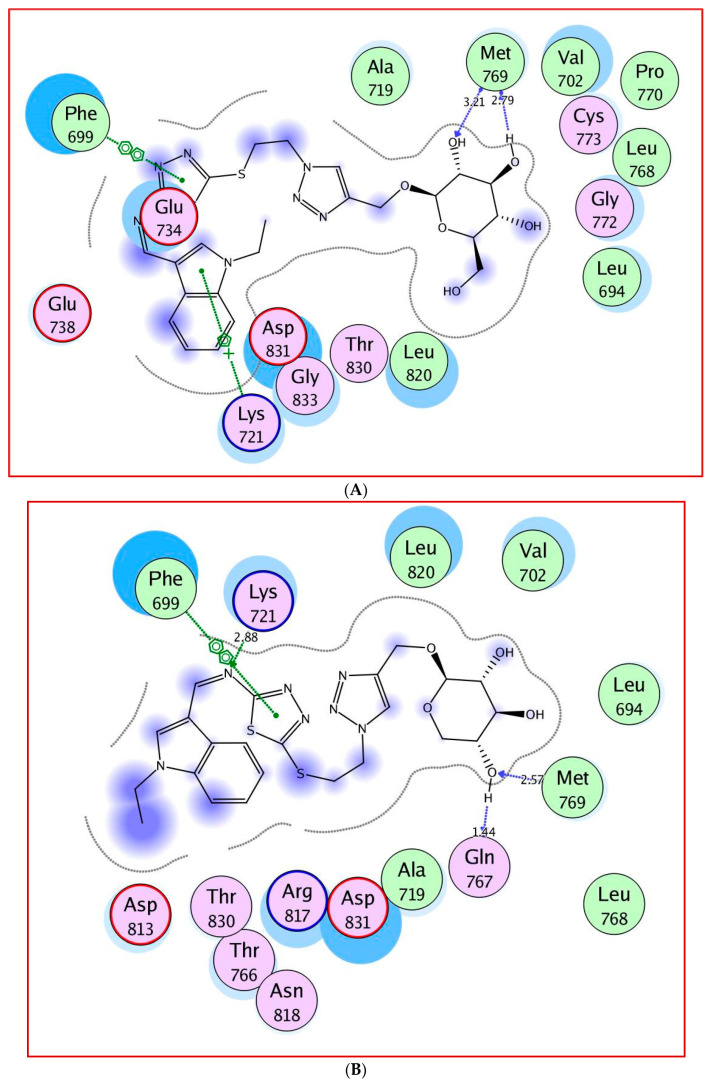

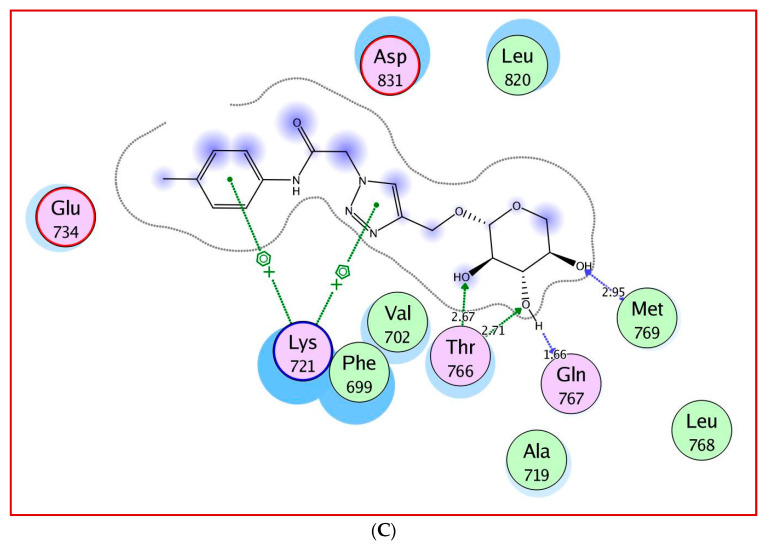
(**A**–**C**) Two-dimensional views of compounds **7**, **8** and **15**, respectively, docked in the active binding site of EGFR^WT^ (PDB ID: 1M17) using MOE software.

**Figure 5:**
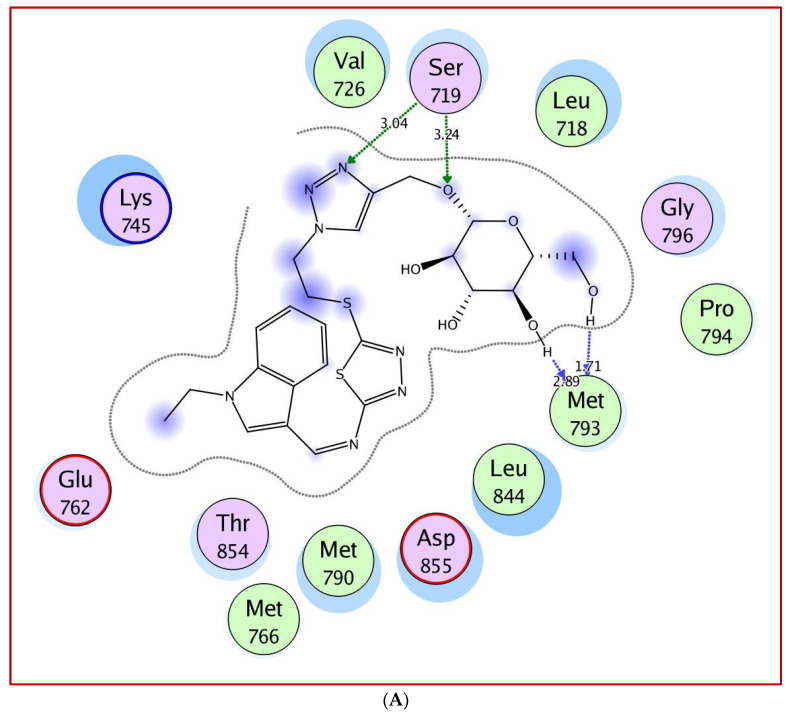

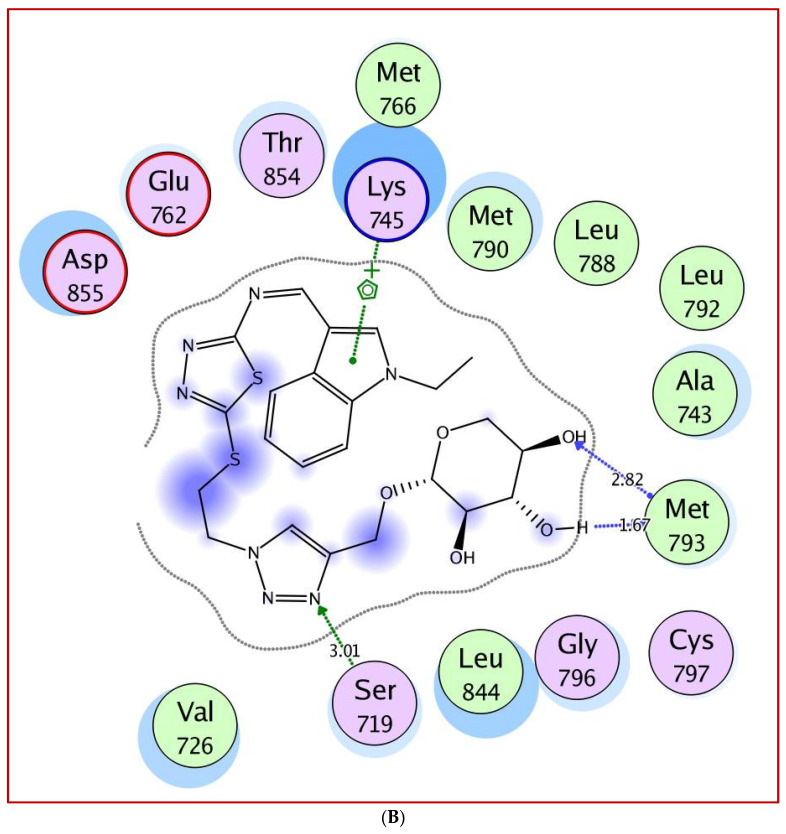

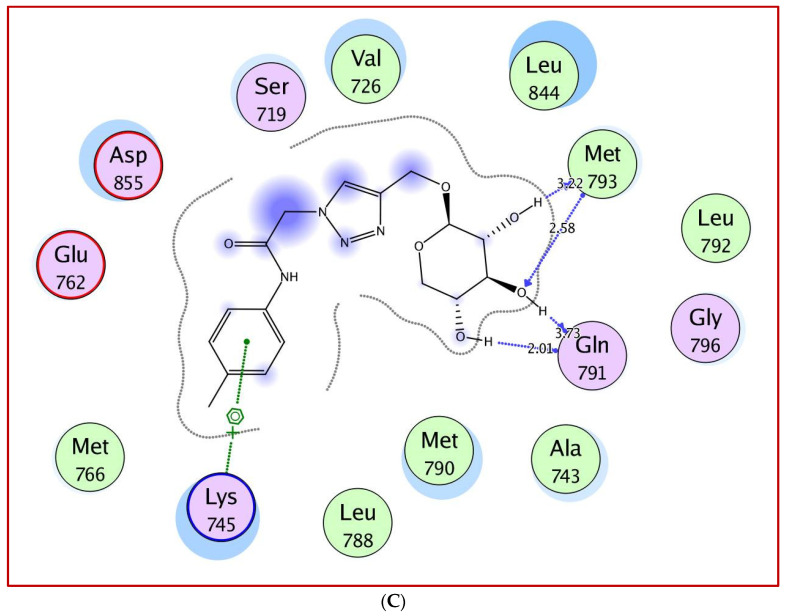
(**A**–**C**) Two-dimensional views of compounds **7**, **8** and **15**, respectively, docked in the active binding site of EGFR^T790M^ (PDB ID: 3UG2) using MOE software.

**Figure 6:**
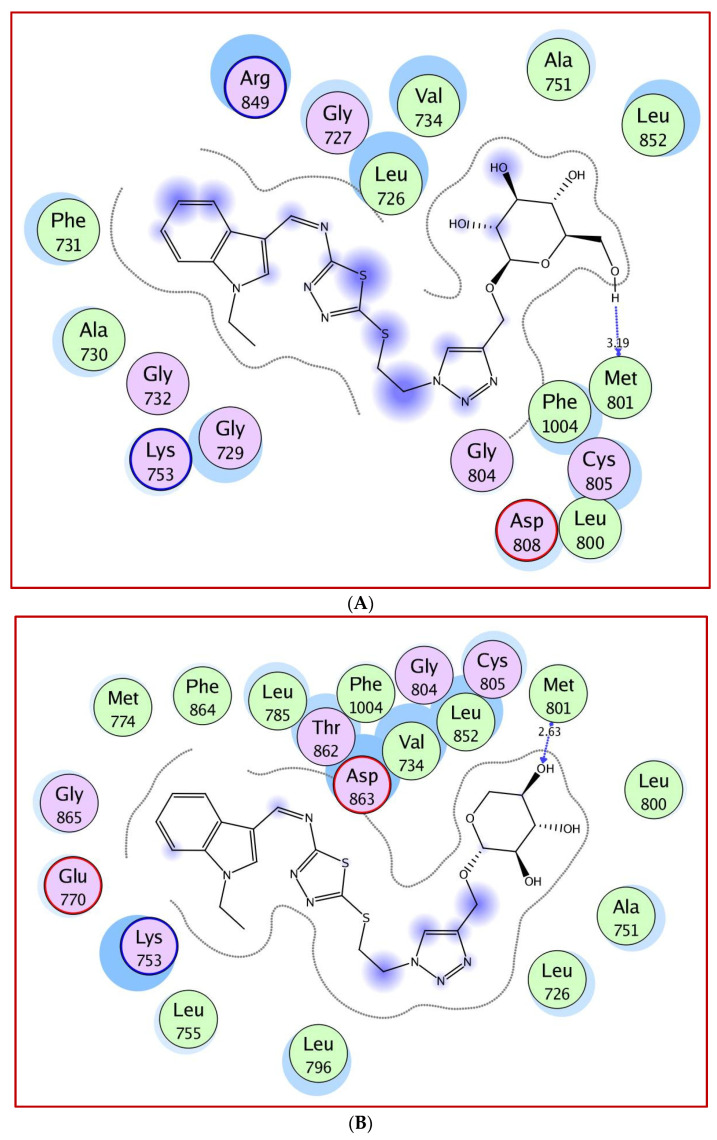

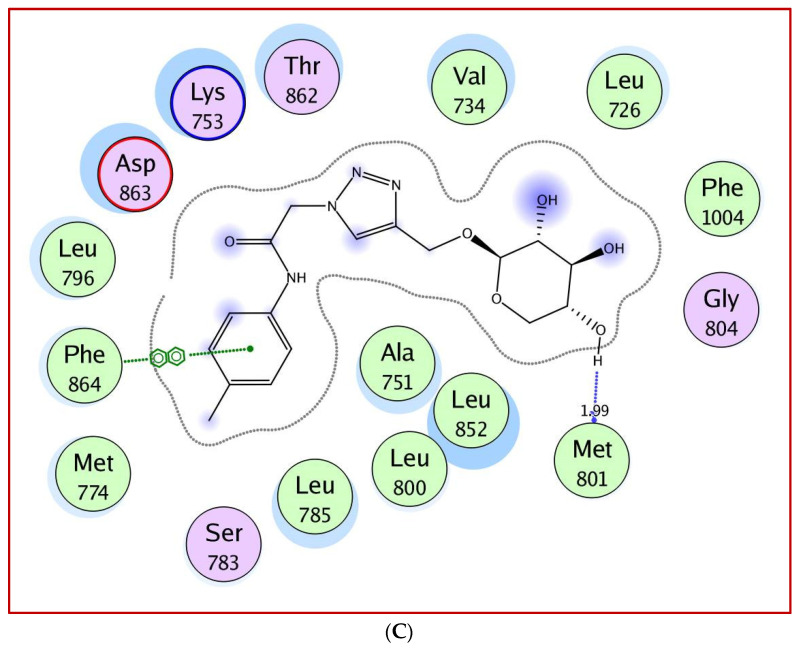
(**A**–**C**) Two-dimensional views of compounds **7**, **8** and **15**, respectively, docked in the active binding site of HER-2 (PDB ID: 3RCD) using MOE software.

As illustrated in the docking of our targets (Figure 4, Figure 5 and Figure 6), the hydroxyl groups of the glycoside scaffolds formed H-bonds with the backbones of **Met769, Met793** and **Met801** within the EGFR^WT^, EGFR^T790M^ and HER-2 binding pockets. Moreover, the glycoside moieties improved fixation within EGFR^WT^ through the formation of more H-bonding with **Gln767** (in **8** and **15**) and **Thr766** (in **15**) and within EGFR^T790M^ through H-bonding with **Gln791**.

Regarding the EGFR^WT^ receptor, the thiadiazole fragments (in **7** and **8**) were observed to play a vital role in binding through arene–arene interactions with **Phe699**. The essential amino acid **Lys721** exhibited arene–cation interactions with the indole moiety in **7** and with p-tolyl and triazole centroids in **15** (Figure 4).

Within EGFR^T790M^ (Figure 5), arene–cation interactions were illustrated between **Lys745** and the indole moiety in **8** and p-tolyl in **15**. However, the triazole nitrogen and oxymethyl oxygen in **7** served as H-bond acceptors with the side chain of **Ser719** (distance: 3.04 and 3.24 Å, respectively).

By focusing on Figure 6, it can be noted that **15** has an improved fit within the active site of HER-2 through an additional arene–arene interaction between the p-tolyl moiety and the essential amino acid **Phe864**.

The superimposition patterns in Figure S6 (in Supplementary Data) demonstrate that the screened derivatives **7**, **8** and **15** are well accommodated, along with the native ligands, erlotinib, gefitinib and TAK-285, within the binding pockets of EGFR^WT^, EGFR^T790M^ and HER-2 through similar H-bonding with the key amino acids **Met769**, **Met793** and **Met801**, respectively. In general, these results highlight targets **7**, **8** and **15** as excellent leads for the discovery of promising anticancer agents through the suppression of EGFR^WT^, EGFR^T790M^ and HER-2 kinases.

## 3. Experimental Section

### 3.1. General

The melting points were recorded through a Reichert Thermovar apparatus and are uncorrected. The infrared spectra were recorded using a Perkin-Elmer (model 1720) FTIR spectrometer. Nuclear magnetic resonance investigations were performed on a Bruker AC-300 or DPX-300 spectrometer (400 MHz ^1^H) (100 MHz ^13^C). The values of δ_ppm_ (chemical shifts) are reported relative to TMS as a standard reference. The J values (coupling constants) are provided in Hz. The progress of the reaction was checked and guided by TLC using aluminum silica gel 60 F245. IR, ^1^H NMR, ^13^C NMR and elemental analyses were measured at the Micro Analytical Center at the Faculty of Science, Cairo University, Egypt.

### 3.2. Synthesis of Bromoethyl-1,3,4-Thidiazole Derivative ***2*** and Azide Derivative ***3***

*N*-(5-((2-Bromoethyl)thio)-1,3,4-thiadiazol-2-yl)-1-(1-ethyl-1H-indol-3-yl)methanimine (**2**).

A mixture of the indolyl-1,3,4-thiadiazole derivative **1** (1.44 g, 5 mmol) and NaOH (0.2 g, 5 mmol) was dissolved in a water–ethanol mixture (1:3, 20 mL) with stirring for about 20 min. The formed mixture was added dropwise to a mixture of dibromopropane (2.81, 15 mmol) and ethanol (7 mL), and stirring was continued for 2 h. The mixture was left to stand for 3 h at room temperature, and the afforded precipitate was filtered and dried to obtain the bromoethyl-1,3,4-thidiazole derivative **2**.

Yield: 75%; m.p. 90–92 °C; IR (KBr) cm^−1^, *ν*: 3290 (alkyne-CH), 3105 (aromatic C-H), 1617 (C=N); ^1^H-NMR (DMSO-d_6_) δ/ppm: 1.42 (t, 3H, *J* = 7.2 Hz, CH_3_), 3.48 (t, 2H, *J* = 6.8, CH_2_), 3.72 (t, 2H, *J* = 6.2 Hz, CH_2_), 4.29 (q, 2H, *J* = 7.2 Hz, CH_2_), 7.30–7.33 (dd, 1H, *J* = 10.4, 8.1 Hz, Ar-H), 7.61–7.64 (d, 1H, *J* = 7.8 Hz, Ar-H), 8.29–8.35 (dd, 2H, *J* = 8.1, 10.6 Hz, Ar-H), 8.87 (s, 1H, indole-H^2^), 9.85 (s, 1H, N=C*H*). ^13^C-NMR (DMSO-d_6_) δ/ppm: 15.0 (CH_3_), 33.2, 33.9 (2CH_2_-N), 41.2 (CH_2_), 111.1, 113.3, 120.8, 122.5, 123.7, 125.0, 130.7, 140.1, 144.9, 145.2 (Ar-C), 162.5 (C=N). Analysis calcd. for C_15_H_15_BrN_4_S_2_ (395.34): C, 45.57; H, 3.82; N, 14.17. Found: C, 45.41; H, 3.91; N, 14.07%.

*N*-(5-((2-Azidoethyl)thio)-1,3,4-thiadiazol-2-yl)-1-(1-ethyl-1*H*-indol-3-yl)methanimine (**3**).

Yield: 85%; m.p. 101–102 °C; IR (KBr) cm^−1^, *ν*: 3289 (alkyne-CH), 3101 (aromatic C-H), 2100 (N=N=N), 1626 (C=N); ^1^H-NMR (DMSO-d_6_) δ/ppm: 1.39 (t, 3H, *J* = 6.8 Hz, CH_3_), 3.45 (t, 2H, *J* = 7.2 Hz, CH_2_), 4.17–4.29 (m, 4H, 2CH_2_), 7.28–7.34 (m, 2H, Ar-H), 7.64 (d, 1H, *J* = 7.8 Hz, Ar-H), 8.20 (d, 1H, *J* = 10.8, 8.1 Hz, Ar-H), 8.86 (s, 1H, indole-H^2^), 9.88 (s, 1H, N=C*H*). ^13^C-NMR (DMSO-d_6_) δ/ppm: 15.1 (CH_3_), 33.1, 33.9 (2CH_2_-N), 41.3 (CH_2_), 111.1, 113.3, 120.7, 122.4, 123.7, 125.1, 130.7, 140.1, 144.9, 145.1 (Ar-C), 162.4 (C=N). Analysis calcd. for C_15_H_15_N_7_S_2_ (357.45): C, 50.40; H, 4.23; N, 27.43. Found: C, 50.56; H, 4.12; N, 27.60.%.

### 3.3. Synthesis of Acetylated O-Glycosides (***5***,***6***)

1-(1-Ethyl-1*H*-indol-3-yl)-*N*-(5-((2-(4-((*O*-acetyl-D-glycopyranosyl)oxymethyl)-1*H*-1,2,3-triazol-1-yl)ethyl)thio)-1,3,4-thiadiazol-2-yl)methanimine (**5**,**6**).

To a well-stirred mixture of the acetylated acetylenic gluco- or xylopyranosyl sugar (2 mmol) and the azidothio thiadiazole derivative **3** (2 mmol) in THF–H_2_O (2:1; 25 mL) was added CuSO_4_·5H_2_O (0.4 mmol), followed by sodium ascorbate (0.4 mmol) and a few drops of diisopropylethylamine at 0 °C. The reaction mixture was stirred at room temperature for 2 h and then at 60 °C for 6 h (TLC, petroleum ether–EtOAc, 3:1). Ethyl acetate (30 mL) was added, and the organic layer was separated, washed with water (2 × 40 mL) and dried. The residue was purified by column chromatography (petroleum ether–EtOAc, 4:1) to afford the acetylated glycosyl triazole compounds **5** and **6**, respectively.

1-(1-Ethyl-1*H*-indol-3-yl)-*N*-(5-((2-(4-({[2,3,4,6-tetra-*O*-acetyl-*β*-D-glucopyranosyl]oxy}methyl)-1*H*-1,2,3-triazol-1-yl)ethyl)thio)-1,3,4-thiadiazol-2-yl)methanimine (**5**).

Yield: 79%; m.p. 131–132 °C; IR (KBr) cm^−1^, *ν*: 3279 (alkyne-CH), 3090 (aromatic C-H), 1738 (C=O), 1612 (C=N); ^1^H-NMR (DMSO-d_6_) δ/ppm: 1.40 (t, 3H, *J* = 7.2 Hz, CH_3_), 1.98, 1.99, 2.01, 2.17 (4s, 12H, CH_3_CO), 3.59 (t, 2H, *J* = 7.2 Hz, CH_2_), 3.72 (m, 3H, CH_2_, H-5′), 4.02 (dd, 1H, *J* = 6.9 Hz, H-6′′), 4.14–4.16 (m, 1H, H-6′), 4.19–4.23 (q, 2H, *J* = 7.2 Hz, CH_2_), 4.25–4.33 (m, 1H, H-4′), 4.99–5.11 (m, 1H, H-2′), 5.34 (t, 1H, H-3′), 4.63 (s, 2H, CH_2_), 6.07 (d, 1H, *J* = 8.4 Hz, H-1′), 7.29 (s, 1H, triazole-H), 7.29 (d, 1H, *J* = 8.1 Hz, Ar-H), 7.62 (d, 1H, *J* = 7.8 Hz, Ar-H), 8.28–8.35 (m, 2H, Ar-H), 8.87 (s, 1H, indole-H^2^), 9.90 (s, 1H, N=C*H*).^13^C-NMR (DMSO-d_6_) δ/ppm: 14.9, 20.2, 20.3, 20.4, 20.6 (5CH_3_), 33.1, 33.9 (2CH_2_-N), 41.3 (CH_2_), 61.2 (CH_2_), 61.2 (C-6), 67.3 (C-4), 68.7 (C-2), 69.1 (C-3), 88.2 (C-5), 90.3 (C-1), 111.1, 113.3, 120.5, 122.5, 123.8, 125.0, 137.2, 140.1, 143.6, 144.4 (Ar-C, triazole-2C,), 162.4 (C=N). 164.2, 169.0, 169.1, 169.5, 169.6, 170.0 (4C=O, thiadiazole-C^2^,C^5^). Analysis calcd. for C_32_H_37_N_7_O_10_S_2_ (743.81): C, 51.67; H, 5.01; N, 13.18. Found: C, 51.48; H, 5.11; N, 13.31.%.

1-(1-Ethyl-1*H*-indol-3-yl)-*N*-(5-((2-(4-({[2,3,4-tri-*O*-acetyl-*β*-D-xylopyranos-yl]oxy}methyl)-1*H*-1,2,3-triazol-1-yl)ethyl)thio)-1,3,4-thiadiazol-2-yl)methanimine (**6**).

Yield: 68%; m.p. 136–138 °C; IR (KBr) cm^−1^, *ν*: 3278 (alkyne-CH), 3092 (aromatic C-H), 1740 (C=O) 1620 (C=N); ^1^H-NMR (DMSO-d_6_) δ/ppm: 1.43 (t, 3H, *J* = 7.1 Hz, CH_3_), 1.97, 1.99, 2.01 (3s, 9H, CH_3_CO), 3.52 (t, *J* = 7.4 Hz, 2H, CH_2_), 3.72 (t, 2H, *J* = 7.4 Hz, CH_2_), 3.93–3.96 (dd, 1H, *J* = 11.6, 2.8 Hz, H-5′′), 4.25 (m, 3H, CH_2_, H-5′), 4.63 (s, 2H, CH_2_), 4.79 (dd, 1H, *J* = 10.6, 7.8 Hz, H-4′), 4.84 (dd, 1H, *J* = 10.2, 7.8 Hz, H-2′), 5.19 (t, *J* = 7.8 Hz, 1H, H-3′), 6.01 (d, 1H, *J* = 10.2 Hz, H-1′), 7.24 (s, 1H, triazole-H), 7.30–7.33 (m, 1H, Ar-H), 7.63–7.66 (m, 1H, Ar-H), 7.90 (d, 1H, *J* = 7.7 Hz, Ar-H), 8.01 (d, 1H, *J* = 8.2 Hz, Ar-H), 8.18 (s, 1H, indole-H^2^), 9.59 (s, 1H, N=C*H*). ^13^C-NMR (DMSO-d_6_) δ/ppm: 14.9, 20.1, 20.3, 20.5 (4CH_3_), 33.2, 33.8 (2CH_2_-N), 41.3 (CH_2_), 61.1 (CH_2_), 67.4 (C-5), 68.6 (C-4), 69.1 (C-2), 88.1 (C-3), 89.9 (C-1), 111.2, 113.5, 120.5, 122.3, 123.9, 125.1, 137.7, 140.1, 143.4, 144.8 (Ar-C, triazole-2C,), 162.4 (C=N). 164.3, 169.1, 169.2, 169.6, 170.1 (3C=O, thiadiazole-C^2^,C^5^). Analysis calcd. for C_29_H_33_N_7_O_8_S_2_ (671.74): C, 51.85; H, 4.95; N, 14.60. Found: C, 51.66; H, 5.03; N, 14.43.%.

### 3.4. Synthesis of Deacetylated O-Glycosides (***7***,***8***)

The acetylated 1,2,3-triazole glycoside **5** or **6** (5 mmol) was added to a saturated methanolic ammonia solution (15 mL) at 0 °C and stirred for 20 min, followed by stirring at room temperature for 7 h. After the completion of the deacetylation process (TLC, petroleum ether–hexane, 2:1), the solvent was evaporated under reduced pressure at 40 °C to afford a yellowish residue. Trituration with cold diethyl ether (20 mL) upon stirring afforded a solid, which was filtered off, dried and crystallized from ethanol to yield compound **7** or **8**, respectively.

1-(1-Ethyl-1*H*-indol-3-yl)-*N*-(5-((2-(4-({[*β*-D-glucopyranosyl]oxy}methyl)-1*H*-1,2,3-triazol-1-yl)ethyl)thio)-1,3,4-thiadiazol-2-yl)methanimine (**7**).

Yield: 65%; m.p. 210–213 °C; IR (KBr) cm^−1^, *ν*: 3455–3425 (OH), 3072 (C-H), 2958 (C-H), 1618 (C=N); ^1^H-NMR (DMSO-d_6_) δ/ppm: 1.41 (t, 3H, *J* = 7.2 Hz, CH_3_), 3.24 (t, 2H, *J* = 7.4 Hz, CH_2_), 3.45–3.49 (m, 2H, H-6′,6′′), 3.66–3.69 (m, 1H, H-5′), 3.87 (t, 2H, CH_2_), 3.96–4.14 (m, 2H, H-4′,3′), 4.29 (q, 2H, *J* = 7.2 Hz, CH_2_), 4.52–4.64 (m, 2H, OH, H-2′), 4.79 (s, 2H, CH_2_), 5.01–5.05 (m, 1H, OH), 5.25–5.28 (m, 1H, OH), 5.35–5.39 (m, 1H, OH), 5.90 (d, 1H, *J* = 10.2 Hz, H-1′), 7.28–7.30 (m, 2H, Ar-H), 7.61 (d, 1H, *J* = 8.1 Hz, Ar-H), 8.08 (d, 1H, *J* = 7.7 Hz, Ar-H), 8.18 (s, 1H, triazole-H), 8.33 (s, 1H, N=C*H*), 9.88 (s, 1H, indole-H^2^). ^13^C-NMR (DMSO-d_6_) δ/ppm: 15.4 (CH_3_), 33.1, 33.9 (2CH_2_-N), 41.3 (CH_2_), 61.2 (CH_2_), 62.1 (C-6), 69.9 (C-4), 72.5 (C-2), 77.4 (C-3), 80.4 (C-5), 87.8 (C-1), 111.4, 117.5, 121.4, 122.9, 123.3, 123.9, 125.1, 137.1, 140.5, 142.8 (Ar-C, triazole-C^4^,C^5^,), 162.4 (C=N), 164.2, 164.3 (thiadiazole-C^2^,C^5^). Analysis calcd. for C_24_H_29_N_7_O_6_S_2_ (575.66): C, 50.08; H, 5.08; N, 17.03. Found: C, 50.23; H, 4.98; N, 17.12%.

1-(1-Ethyl-1*H*-indol-3-yl)-*N*-(5-((2-(4-({[β-*D*-Xylopyranosyl]oxy}methyl)-1*H*-1,2,3-triazol-1-yl)ethyl)thio)-1,3,4-thiadiazol-2-yl)methanimine (**8**).

Yield: 68%; m.p. 203–204 °C; IR (KBr) cm^−1^, *ν*: 3455–3425 (OH), 3070 (C-H), 1618 (C=N); ^1^H-NMR (DMSO-d_6_) δ/ppm: 1.41 (t, 3H, *J* = 7.0 Hz, CH_3_), 3.42 (t, 2H, *J* = 7.4 Hz, CH_2_), 3.69 (dd, 1H, *J* = 11, 5.3 Hz, H-5′′), 4.05–4.15 (m, 3H, H-5′, CH_2_), 4.17–4.22 (m, 1H, H-4′), 4.29 (q, 2H, *J* = 7.0 Hz, CH_2_), 4.63 (s, 2H, CH_2_), 4.87–4.91 (m, 2H, H-2′, OH), 4.91–4.94 (m, 1H, OH), 5.12–5.15 (m, 1H, OH), 5.29 (dd, 1H, *J* = 7.7, 5.0 Hz, H-3′), 5.90 (d, 1H, *J* = 5.1 Hz, H-1′), 7.24–7.27 (m, 1H, Ar-H), 7.30–7.33 (m, 1H, Ar-H), 7.61 (d, 1H, *J* = 8.1 Hz, Ar-H), 8.09 (d, 1H, *J* = 7.6 Hz, Ar-H), 8.21 (s, 1H, triazole-H), 8.33 (s, 1H, indole-H^2^), 9.88 (s, 1H, N=C*H*). ^13^C-NMR (DMSO-d_6_) δ/ppm: 15.4 (CH_3_), 33.1, 33.9 (2CH_2_-N), 41.3 (CH_2_), 61.2 (CH_2_), 69.7 (C-4), 72.8 (C-2), 77.6 (C-3), 80.5 (C-5), 87.2 (C-1), 111.2, 117.8, 121.5, 122.9, 123.1, 123.9, 125.5, 137.3, 140.6, 142.8 (Ar-C, triazole-C^4^,C^5^), 162.4 (C=N), 164.2, 164.3 (thiadiazole-C^2^,C^5^). Analysis calcd. for C_23_H_27_N_7_O_5_S_2_ (545.63): C, 50.63; H, 4.99; N, 17.97. Found: C, 50.46; H, 5.11; N, 18.09%.

2-(4-({[*O*-Acetyl-*β*-D-glycopyranosyl]oxy}methyl)-1*H*-1,2,3-triazol-1-yl)-*N*-(aryl)acetamide (**11**–**14**).

### 3.5. Synthesis of Acetylated O-Glycosides ***11***–***14***

The same procedure for the preparation of **5** and **6** was applied. To a well-stirred mixture of the acetylated acetylenic xylo- or galactopyranosyl sugar (2 mmol) and the azide **9a,b** (2 mmol) in THF–H_2_O (2:1; 25 mL) was added CuSO_4_·5H_2_O (0.4 mmol), followed by sodium ascorbate (0.4 mmol) and a few drops of diisopropylethylamine at 0 °C. The reaction mixture was stirred at room temperature for 1 h and then at 60 °C for 8 h (TLC, petroleum ether–EtOAc, 3:1). Ethyl acetate (35 mL) was added, and the organic layer was separated, washed with water (2 × 45 mL) and dried. The residue was purified by column chromatography (petroleum ether–EtOAc, 5:1) to afford the acetylated 1,2,3-triazole-glycosides **11**–**14**, respectively.

2-(4-({[2,3,4-Tri-*O*-acetyl-*β*-D-xylopyranosyl]oxy}methyl)-1*H*-1,2,3-triazol-1-yl)-*N*-(p-tolyl)acetamide (**11**).

Yield: 71%; m.p. 95–96 °C; IR (KBr) cm^−1^, *ν*: 3150 (NH), 3094 (aromatic C-H), 1742 (C=O), 1668 (C=O), 1618 (C=N); ^1^H-NMR (DMSO-d_6_) δ/ppm: 2.00, 2.02, 2.04 (3s, 9H, CH_3_CO), 2.29 (s, 3H, CH_3_), 4.14 (dd, 1H, *J* = 11.7, 5.4 Hz, H-5′′), 4.67 (dd, 1H, *J* = 6.6, 11.7 Hz, H-5′), 4.33 (s, 2H, CH_2_), 4.77 (dd, 1H, *J* = 22.9, 9.4 Hz, H-4′), 4.95–4.98 (m, 1H, H-2′), 5.27 (t, 1H, *J* = 8.3 Hz, H-3′), 5.47 (s, 2H, CH_2_), 5.97 (d, 1H, *J* = 3.6 Hz, H-1′), 7.11 (d, 2H, *J* = 8.2 Hz, Ar-H), 7.33 (d, 2H, *J* = 8.2 Hz, Ar-H), 8.06 (s, 1H, triazole), 10.01 (s, 1H, N*H*). ^13^C-NMR (DMSO-d_6_) δ/ppm: 20.2, 20.3, 20.4, 20.5 (4CH_3_), 51.3, 61.4 (2CH_2_), 66.3 (C-5), 66.6 (C-4), 67.2 (C-2), 68.1 (C-3), 88.6 (C-1), 119.2, 119.3, 129.1, 129.2, 133.6, 134.4, 135.8, 142.1 (Ar-C, triazole-C^4^,C^5^), 164.9, 169.4, 169.7, 169.9 (4C=O). Analysis calcd. for C_23_H_28_N_4_O_9_ (504.50): C, 54.76; H, 5.59; N, 11.11. Found: C, 54.63; H, 5.51; N, 11.19.%.

2-(4-({[2,3,4,6-Tetra-*O*-acetyl-*β*-D-galactopyranosyl]oxy}methyl)-1*H*-1,2,3-triazol-1-yl)-*N*-(p-tolyl)acetamide (**12**).

Yield: 64%; m.p. 98–99 °C; IR (KBr) cm^−1^, *ν*: 3155 (NH), 3093 (aromatic C-H), 1739 (C=O), 1668 (C=O), 1620 (C=N); ^1^H-NMR (DMSO-d_6_) δ/ppm: 1.96, 1.98, 2.00, 2.02 (4s, 12H, CH_3_CO), 2.25 (s, 3H, CH_3_), 3.72–4.75 (m, 1H, H-5′), 4.00–4.03 (m, 1H, H-6′′), 4.04–4.07 (m, 1H, H-6′), 4.63 (s, 2H, CH_2_), 5.09–5.14 (m, 1H, H-4′), 5.27–5.31 (dd, 1H, *J* = 9.4, *J* = 10.2, H-2′), 5.43 (t, 1H, *J* = 9.4 Hz, H-3′), 5.44 (s, 2H, CH_2_), 6.02 (d, 1H, *J* = 10.2 Hz, H-1′), 7.12 (d, 2H, *J* = 8.2 Hz, Ar-H), 7.46 (d, 2H, *J* = 8.2 Hz, Ar-H), 8.06 (s, 1H, triazole), 10.01 (brs, 1H, N*H*). ^13^C-NMR (DMSO-d_6_) δ/ppm: 20.1, 20.2, 20.3, 20.4, 20.5 (5CH_3_), 51.1, 61.2 (2CH_2_), 66.1 (C-6), 66.2 (C-5), 66.7 (C-4), 67.3 (C-2), 68.3 (C-3), 88.7 (C-1), 119.1, 119.2, 129.1, 129.2, 132.6, 134.5, 135.8, 142.3 (Ar-C, triazole-2C,), 165.9, 169.0, 169.4, 169.7, 169.9 (5C=O). Analysis calcd. for C_26_H_32_N_4_O_11_ (576.56): C, 54.16; H, 5.59; N, 9.72. Found: C, 54.29; H, 5.49; N, 9.86.%.

2-(4-({[2,3,4-Tri-*O*-acetyl-*β*-D-xylopyranosyl]oxy}methyl)-1*H*-1,2,3-triazol-1-yl)-*N*-(4-fluorophenyl)acetamide (**13**).

Yield: 73%; m.p. 142–144 °C; IR (KBr) cm^−1^, *ν*: 3148 (NH), 3094 (aromatic C-H), 1738 (C=O), 1668 (C=O), 1615 (C=N); ^1^H-NMR (DMSO-d_6_) δ/ppm: 1.97, 1.98, 2.13 (3s, 9H, CH_3_CO), 4.14 (dd, 1H, *J* = 10.6, 3.8 Hz, H-5′′), 4.37 (dd, 1H, *J* = 3.8, 9.8 Hz, H-5′), 4.67 (s, 2H, CH_2_-O), 4.76 (dd, 1H, *J* = 8.5, 9.8 Hz, H-4′), 4.77- 4.82 (dd, 1H, *J* = 10.2, 8.8 Hz, H-2′), 5.17 (t, *J* = 8.8 Hz, 1H, H-3′), 5.29 (s, 2H, CH_2_-N), 6.16 (d, 1H, *J* = 10.2 Hz, H-1′), 7.14 (d, 2H, *J* = 8.0 Hz, Ar-H), 7.55 (d, 2H, *J* = 8.2 Hz, Ar-H), 8.06 (s, 1H, triazole), 10.52 (s, 1H, N*H*). ^13^C-NMR (DMSO-d_6_) δ/ppm: 20.0, 20.1, 20.3 (3CH_3_), 51.9, 61.4 (2CH_2_), 67.7 (C-5), 68.4 (C-4), 70.2 (C-2), 77.5 (C-3), 99.1 (C-1), 115.2, 120.3, 121.2, 125.7, 130.3, 134.4, 142.9, 159.8 (Ar-C, triazole-2C,), 163.9, 169.1, 169.6, 169.7 (4C=O). Analysis calcd. for C_22_H_25_FN_4_O_9_ (508.46): C, 51.97; H, 4.96; N, 11.02. Found: C, 52.11; H, 5.09; N, 10.88.%.

2-(4-({[2,3,4,6-Tetra-*O*-acetyl-*β*-D-galactopyranosyl]oxy}methyl)-1*H*-1,2,3-triazol-1-yl)-*N*-(4-fluorophenyl)acetamide (**14**).

Yield: 77%; m.p. yellowish foam; IR (KBr) cm^−1^, *ν*: 3190 (NH), 3099 (aromatic C-H), 1740 (C=O) 1666 (C=O), 1616 (C=N); ^1^H-NMR (DMSO-d_6_) δ/ppm: 1.98, 1.99, 2.01, 2.11 (4s, 12H, CH_3_CO), 3.70–3.73 (m, 1H, H-5′), 4.03 (dd, 1H, *J* = 11.6, 2.8 Hz, H-6′′), 4.09–4.11 (m, 1H, H-6′), 4.67 (s, 2H, CH_2_-O), 4.70–4.74 (m, 1H, H-4′), 4.82–4.85 (dd, 1H, *J* = 10.2, 8.8 Hz, H-2′), 5.26 (t, 1H, *J* = 8.8 Hz, H-3′), 5.32 (s, 2H, CH_2_-N), 6.02 (d, 1H, *J* = 10.2 Hz, H-1′), 7.17 (d, 2H, *J* = 8.2 Hz, Ar-H), 7.59 (d, 2H, *J* = 8.2 Hz, Ar-H), 8.08 (s, 1H, triazole), 10.50 (s, 1H, N*H*). ^13^C-NMR (DMSO-d_6_) δ/ppm: 20.1, 20.2, 20.3, 20.4 (4CH_3_), 51.8, 61.6 (2CH_2_), 66.4 (C-6), 67.9 (C-5), 68.3 (C-4), 70.1 (C-2), 77.8 (C-3), 99.1 (C-1), 115.5, 120.1, 121.7, 125.7, 130.3, 134.8, 142.9, 159.8 (Ar-C, triazole-C^4^,C^5^), 164.2 (C=O). 169.4, 169.6, 169.7, 169.9 (C=O). Analysis calcd. for C_25_H_29_FN_4_O_11_ (580.52): C, 51.72; H, 5.03; N, 9.65. Found: C, 51.56; H, 4.91; N, 9.79.%.

### 3.6. Synthesis of Deacetylated O-Glycosides ***15***–***18***

2-(4-({[*β*-D-Xylopyranosyl]oxy}methyl)-1*H*-1,2,3-triazol-1-yl)-*N*-(p-tolyl)acetamide (**15**).

Yield: 76%; m.p. 193–194 °C; IR (KBr) cm^−1^, *ν*: 3438–3455 (OH), 3200 (NH), 3094 (aromatic C-H), 1660 (C=O), 1615 (C=N); ^1^H-NMR (DMSO-d_6_) δ/ppm: 2.24 (s, 3H, CH_3_), 3.34–3.37 (m, 2H, H-5′′, H-5′), 3.92–3.95 (m, 1H, H-4′), 4.28- 4.31 (m, 1H, H-3′), 4.35 (s, 2H, CH_2_), 4.88 (m, 2H, H-2′, OH), 4.98–5.01 (m, 1H, OH), 5.14–5.17 (m, 1H, OH), 5.30 (s, 2H, CH_2_), 5.72 (d, 1H, *J* = 3.6 Hz, H-1′), 7.12 (d, 2H, *J* = 8.2 Hz, Ar-H), 7.46 (d, 2H, *J* = 8.2 Hz, Ar-H), 8.11 (s, 1H, triazole), 10.40 (s, 1H, N*H*). ^13^C-NMR (DMSO-d_6_) δ/ppm: 20.5 (CH_3_), 52.2, 61.5 (2CH_2_), 65.8 (C-5), 69.6 (C-4), 73.3 (C-2), 76.5 (C-3), 102.9 (C-1), 119.2, 119.3, 125.9, 129.3, 132.8, 135.9, 143.5, 164.1 (Ar-C, triazole-C^4^,C^5^), 171.8 (C=O). Analysis calcd. for C_17_H_22_N_4_O_6_ (378.39): C, 53.96; H, 5.86; N, 14.81. Found: C, 54.09; H, 5.80; N, 15.01.%.

2-(4-({[*β*-D-Galactopyranosyl]oxy}methyl)-1*H*-1,2,3-triazol-1-yl)-*N*-(p-tolyl)acetamide (**16**).

Yield: 75%; m.p. 199–200 °C; IR (KBr) cm^−1^, *ν*: 3448–3430 (OH), 3198 (NH), 3095 (aromatic C-H), 1660 (C=N); ^1^H-NMR (DMSO-d_6_) δ/ppm: 2.25 (s, 3H, CH_3_), 3.39–3.42 (m, 2H, H-6′′, H-6′), 3.79–3.81 (m, 1H, H-5′), 4.18–4.33 (m, 3H, CH_2_, H-4′), 4.77–4.81 (m, 2H, H-3′, OH), 4.99–5.08 (m, 2H, H-2′, OH), 5.12–5.15 (m, H, OH), 5.25–5.28 (m, 3H, OH, CH_2_), 5.80 (d, 1H, *J* = 10.2 Hz, H-1′), 7.12 (d, 2H, *J* = 8.2 Hz, Ar-H), 7.45 (d, 2H, *J* = 8.2 Hz, Ar-H), 8.11 (s, 1H, triazole), 10.40 (s, 1H, N*H*). ^13^C-NMR (DMSO-d_6_) δ/ppm: 21.3 (CH_3_), 52.1, 61.4 (2CH_2_), 64.5 (C-6), 66.8 (C-5), 68.6 (C-4), 74.3 (C-2), 76.5 (C-3), 103.9 (C-1), 119.1, 119.8, 126.9, 129.3, 133.8, 135.9, 144.5, 165.1 (Ar-C, triazole-C^4^,C^5^), 170.8 (C=O). Analysis calcd. for C_18_H_24_N_4_O_7_ (408.41): C, 52.94; H, 5.92; N, 13.72. Found: C, 53.17; H, 6.11; N, 13.51.%.

2-(4-({[*β*-D-Xylopyranosyl]oxy}methyl)-1*H*-1,2,3-triazol-1-yl)-*N*-(4-fluorophenyl)acetamide (**17**).

Yield: 51%; m.p. 197–198 °C; IR (KBr) cm^−1^, *ν*: 3480–3465 (OH), 3210 (NH), 3097 (aromatic C-H), 1661 (C=O), 1612 (C=N); ^1^H-NMR (DMSO-d_6_) δ/ppm: 3.37–3.41 (m, 2H, H-5′′, H-5′), 3.90–3.93 (m, 1H, H-4′), 4.22–4.25 (m, 1H, H-3′), 4.32 (s, 2H, CH_2_), 4.85–4.88 (m, 1H, H-2′), 4.98–5.02 (m, 1H, OH), 5.12–5.15 (m, 1H, OH), 5.29 (s, 2H, CH_2_), 5.37–5.40 (m, 1H, OH), 5.77 (d, 1H, *J* = 9.8 Hz, H-1′), 7.14 (d, 2H, *J* = 8.2 Hz, Ar-H), 7.57 (d, 2H, *J* = 8.2 Hz, Ar-H), 8.09 (s, 1H, triazole), 10.52 (s, 1H, N*H*). ^13^C-NMR (DMSO-d_6_) δ/ppm: 51.9, 61.3 (2CH_2_), 65.5 (C-5), 69.4 (C-4), 73.1 (C-2), 76.3 (C-3), 102.7 (C-1), 115.1, 121.1, 125.7, 134.5, 143.4, 156.6, 159.8, 164.1 (Ar-C, triazole-C^4^,C^5^), 172.1 (C=O). Analysis calcd. for C_16_H_19_FN_4_O_6_ (382.35): C, 50.26; H, 5.01; N, 14.65. Found: C, 50.39; H, 4.89; N, 14.54.%.

2-(4-({[*β*-*D*-Galactopyranosyl]oxy}methyl)-1*H*-1,2,3-triazol-1-yl)-*N*-(4-fluorophenyl)acetamide (**18**).

Yield: 45%; m.p. Brownish foam; IR (KBr) cm^−1^, *ν*: 3455–3430 (OH), 3200 (NH), 3093 (aromatic C-H), 1663 (C=O), 1615 (C=N); ^1^H-NMR (DMSO-d_6_) δ/ppm: 3.37–3.41 (m, 2H, H-6′′, H-6′), 3.88–3.91 (m, 1H, H-5′), 3.99–4.02 (m, 1H, H-4′), 4.18- 4.22 (m, 1H, H-3′), 4.34 (s, 2H, CH_2_), 4.79–4.89 (m, 2H, OH, H-2′), 4.99–5.02 (m, 1H, OH), 5.25–5.30 (m, 2H, 2OH), 5.30 (s, 2H, CH_2_), 5.80 (d, 1H, *J* = 9.8 Hz, H-1′), 7.13 (d, 2H, *J* = 8.2 Hz, Ar-H), 7.46 (d, 2H, *J* = 8.2 Hz, Ar-H), 8.10 (s, 1H, triazole), 10.50 (s, 1H, N*H*). ^13^C-NMR (DMSO-d_6_) δ/ppm: 51.8, 61.6 (2CH_2_), 65.5 (C-6), 67.9 (C-5), 69.4 (C-4), 72.2 (C-2), 77.4 (C-3), 103.1 (C-1), 116.2, 120.5, 121.9, 126.7, 131.3, 133.4, 144.9, 159.7 (Ar-C, triazole-C^4^,C^5^), 164.9 (C=O). Analysis calcd. for C_17_H_21_FN_4_O_7_ (412.37): C, 49.51; H, 5.13; N, 13.59. Found: C, 49.35; H, 5.20; N, 13.68.%.

## 4. Materials and Methods

### 4.1. In Vitro Cytotoxic Activity

HCT-116 (human colorectal carcinoma) and MCF-7 (human breast adenocarcinoma) cell lines were purchased from the American Type Culture Collection (Rockville, MD, USA) and maintained in Dulbecco’s Modified Eagle’s Medium (DMEM) supplemented with 10% heat-inactivated fetal bovine serum (FBS), 100 U mL^−1^ penicillin and 100 U mL^−1^ streptomycin. The cells were grown at 37 °C in a humidified atmosphere of 5% CO_2_.

### 4.2. MTT Cytotoxicity Assay

Cytotoxicity activity against HCT-116 and MCF-7 was estimated by the 3-[4,5-dimethyl-2-thiazolyl)-2,5-diphenyl-2H-tetrazolium bromide (MTT) assay. This test is based on MTT cleavage by mitochondrial dehydrogenases from viable cells [50,51,52]. Cells were placed in a 96-well sterile microplate (5 × 10^4^ cells well^−1^) and incubated at 37 °C in serum-free media containing dimethyl sulfoxide (DMSO) and either a series of various concentrations of each compound or doxorubicin (positive control) for 48 h before the MTT assay. After incubation, the media were removed, and 40 µL of MTT (2.5 mg mL^−1^) was added to each well. Incubation was resumed for an additional 4 h. The purple formazan dye crystals were solubilized with 200 µL DMSO. Absorbance was measured at 590 nm in a Spectra Max Paradigm Multi-Mode microplate reader (Molecular Devices, LLC, San Jose, CA, USA). Relative cell viability was expressed as the mean percentage of viable cells compared to the untreated control cells.

### 4.3. Statistical Analysis

All experiments were conducted in triplicate and repeated on three different days. All values are reported as mean ± SD. IC_50_ values were determined by SPSS Inc. probit analysis (IBM Corp., Armonk, NY, USA).

### 4.4. EGFR^WT^, EGFR^T790M^ and HER-2 Inhibition Assessment

The promising cytotoxic targets **7, 8, 11**, **12** and **15** were assessed for their in vitro inhibitory activity against EGFR^WT^, EGFR^T790M^ and HER-2 kinases. Erlotinib was used as a reference following the previously described method [53]. A homogeneous time-resolved fluorescence (HTRF) assay was applied in this test (Sigma). Firstly, EGFR^WT^, EGFR^T790M^ and HER-2 and their substrates were incubated with the tested compounds in enzymatic buffer for 5 min. ATP (1.65 μM) was added to the reaction mixture to start the enzymatic reaction. The assay was conducted for 30 min at room temperature. The reaction was stopped by the addition of detection reagents that contained EDTA. The detection step continued for 1 h, and then the IC_50_ values were determined using GraphPad Prism 5.0. Three independent experiments were performed for each concentration.

### 4.5. Detection of Apoptosis and Cell Cycle Analysis

Apoptosis investigation and cell cycle analysis were performed by flow cytometry [43,44]. MCF-7 cells were seeded at 8 × 10^4^ and incubated at 37 °C in 5% CO_2_ overnight. After treatment with the tested compounds **7** and **15** for 24 h, cell pellets were collected and centrifuged (300 g, 5 min). For cell cycle analysis, cell pellets were fixed with 70% ethanol on ice for 15 min and collected again. The pellets were incubated with propidium iodide (PI) staining solution at room temperature for 1 h and analyzed with a Gallios flow cytometer (Beckman Coulter, Brea, CA, USA). Apoptosis detection was carried out by the FITC AnnexinV/PI commercial kit (Becton Dickenson, Franklin Lakes, NJ, USA) following the manufacturer’s protocol. The samples were analyzed by fluorescence-activated cell sorting (FACS) with a Gallios flow cytometer (Beckman Coulter, Brea, CA, USA) within 1 h after staining. Data were analyzed using Kaluza v 1.2 (Beckman Coulter).

## 5. Molecular Docking Study

The interactions of the newly synthesized targets **7, 8** and **15**, illustrating the highest EGFR^WT^, EGFR^T790M^ and HER-2 inhibitory activities, were examined and docked within the active sites of the target enzymes to study their binding modes and orientations using PDB IDs: 1M17, 3UG2 and 3RCD, respectively [47,49], using MOE-Dock software version 2014.0901 [45,46]. The 2D structures of the newly synthesized triazole-based glycosides **7**, **8** and **15** were drawn using Chem. Draw. The protonated 3D structure was employed using standard bond lengths and angles. Then, geometry optimization and energy minimization were applied to obtain the Conf Search module in MOE, and the MOE file was then saved for the subsequent docking process. The co-crystallized structures of EGFR^WT^, EGFR^T790M^ and HER-2 with their ligands, erlotinib, gefitinib and TAK-285, were downloaded (PDB codes: 1M17, 3UG2 and 3RCD, respectively) from the Protein Data Bank. All minimizations were performed using MOE until reaching an RMSD gradient of 0.05 kcal∙mol^−1^Å^−1^ with an MMFF94x force field, and the partial charges were automatically calculated. The preparation of the enzyme structure was performed for molecular docking using the Protonate 3D protocol with the default options in MOE. The London dG scoring function and Triangle Matcher placement method were used in the docking protocol. First, validation of the docking processes was established by docking the native ligands, followed by docking derivatives **7**, **8** and **15** within the ATP-binding sites of EGFR^WT^, EGFR^T790M^ and HER-2 after the elimination of the co-crystallized ligands.

## Figures and Tables

**Table 1:** IC_50_ of the synthesized compounds against the two cancer cell lines according to the MTT assay.

Compound No.	IC_50_ (µM) ± SD	
	HCT-116	MCF-7
**1**	46.9 ± 0.5	18.1 ± 0.2
**2**	52.7 ± 0.6	14.1 ± 0.3
**3**	30.8 ± 0.4	1.1 ± 0.1
**5**	22.6 ± 0.2	13.9 ± 0.2
**6**	20.8 ± 0.2	10.8 ± 1.5
**7**	15.5 ± 0.2	0.5 ± 0.0
**8**	4.6 ± 0.1	4.2 ± 0.1
**9a**	42.3 ± 0.6	24.1 ± 0.3
**9b**	33.3 ± 0.4	26.4 ± 0.3
**11**	15.4 ± 0.2	0.8 ± 0.0
**12**	2.2 ± 0.1	5.7 ± 0.1
**13**	38.2 ± 0.5	34.4 ± 0.4
**14**	33.8 ± 0.3	35.3 ± 0.3
**15**	11.4 ± 0.3	0.6 ±0.0
**16**	35.7 ± 0.4	20.3 ± 0.9
**17**	26.5 ± 0.3	30.1 ± 0.4
**18**	28.8 ± 0.1	31.6 ± 0.5
**Doxorubicin**	12.1 ± 0.2	9.4 ± 0.1

**IC_50_:** The compound concentration that inhibits the proliferation of cells by 50%; SD: mean of the standard deviation; each value is the mean of three values.

**Table 2:** Kinase inhibition assessment of the highly potent derivatives **7**, **8**, **11**, **12** and **15** (IC_50_ in μM) against EGFR^WT^, EGFR^T790M^ and HER-2 enzymes.

Compounds	IC_50_ (Mean ± SEM) (µM)		
	EGFR^WT^	EGFR^T790M^	HER-2
**7**	0.30 ± 0.01	1.12 ± 0.55	2.25 ± 0.30
**8**	0.38 ± 0.05	1.95 ± 0.02	2.77 ± 0.13
**11**	6.45 ± 0.10	11.80 ± 0.05	8.60 ± 0.20
**12**	12.70 ± 0.15	15.22 ± 0.20	18. 40 ± 0.12
**15**	0.21 ± 0.05	1.50 ± 0.11	2.18 ± 0.42
**Erlotinib**	0.13 ± 0.01	0.62 ± 0.10	1.40 ± 0.05

**IC_50_:** The compound concentration required to inhibit the enzyme viability by 50%; SEM = standard error mean; each value is the mean of three values.

**Table 3:** Apoptosis and necrosis data on MCF-7 cells induced by the most potent derivatives **7** and **15**.

Compd.	Apoptosis			Necrosis
	Total	Early	Late	
**7/MCF-7**	34.61	4.39	18.81	11.41
**15/MCF-7**	42.45	2.69	25.48	14.28
**Cont./MCF-7**	1.99	0.46	0.28	1.25

## Data Availability

Not applicable.

## Supplementary Materials

The following supporting information can be downloaded at: https://www.mdpi.com/article/10.3390/molecules27206960/s1, include spectral and biological data of synthesized compound. Figure S1. Dose dependent cytotoxic activity of the synthesized derivatives against HCT-116 cancer cells according to the MTT assay. Figure S2. Dose dependent cytotoxic activity of the synthesized derivatives against MCF-7 cancer cells according to the MTT assay. Figure S3. IC50 (µM) data of the screened targets in comparison with doxorubicin against human HCT-116 and MCF7 cancer cell lines according to the MTT assay. Figure S4. Apoptotic activity of the promising derivatives **7** and **15**. Figure S5. Cell cycle analysis in MCF-7 cells treated with the promising derivatives **7** and **15**. Figure S6. 3D diagrams of the superimposition of the original ligands erlotinib, gefitinib and TAK-285 (red), **7** (blue), **8** (yellow) and **15** (green), within the ATP-binding pockets of EGFRWT, EGFRT790M and HER-2 (PDB codes: 1M17, 3UG2 and 3RCD), respectively. ^1^H-NMR and ^13^ C-NMR for compound **2**,**3**,**5**,**6**,**7**,**8**,**11**,**12**,**13**,**14**.

## References

[1] Avin B.R.V., Thirusangu P., Ranganatha V.L., Firdouse A., Prabhakar B.T., Khanum S.A. (2014). Synthesis and tumor inhibitory activity of novel coumarin analogs targeting angiogenesis and apoptosis. Eur. J. Med. Chem..

[2] Jiang N., Zhai X., Zhao Y., Liu Y., Qi B., Tao H., Gong P. (2012). Synthesis and biological evaluation of novel 2-(2-arylmethylene)hydrazinyl-4-aminoquinazoline derivatives as potent antitumor agents. Eur. J. Med. Chem..

[3] Jain A.K., Sharma S., Vaidya A., Ravichandran V., Agrawal R.K. (2013). 1, 3, 4-thiadiazole and its derivatives: A review on recent progress in biological activities. Chem. Biol. Biol. Biol. Biol. Drug Des..

[4] Kushwaha N., Kushwaha S.K.S., Rai A.K. (2012). Biological activities of thiadiazole derivatives: A review. Int. J. Chem. Res..

[5] Gomha S.M., Salah T.A., Abdelhamid A.O. (2015). Synthesis, characterization, and pharmacological evaluation of some novel thiadiazoles and thiazoles incorporating pyrazole moiety as anticancer agents. Monatsh. Chem..

[6] Gomha S.M., Abdel-aziz H.M. (2015). Synthesis and antitumor activity of 1,3,4-thiadiazole derivatives bearing coumarine ring. Heterocycles.

[7] Siddiqui N., Ahuja P., Ahsan W., Pandeya S.N., Alam M.S. (2009). Thiadiazoles: Progress report on biological activities. J. Chem. Pharm. Res..

[8] Foroumadi A., Kargar Z., Sakhteman A., Sharifzadeh Z., Feyzmohammadi R., Kazemi M., Shafiee A. (2006). Synthesis and antimycobacterial activity of some alkyl [5-(nitroaryl)-1,3,4-thiadiazol-2-ylthio]propionates. Bioorganic Med. Chem. Lett..

[9] Kumar D., Kumar N.M., Chang K.H., Shah K. (2010). Synthesis and anticancer activity of 5-(3-indolyl)-1,3,4-thiadiazoles. Eur. J. Med. Chem..

[10] Ay K., Ispartaloglu B., Halay E., Ay E., Yasa I., Karayıldırım T. (2017). Synthesis and antimicrobial evaluation of sulfanilamide- and carbohydrate-derived 1,4-disubstitued-1,2,3-triazoles via click chemistry. Med. Chem. Res..

[11] Subhashini N.J.P., Kumar E.P., Gurrapu N., Yerragunta V. (2019). Design and synthesis of imidazolo-1,2,3-triazoles hybrid compounds by microwave-assisted method: Evaluation as an antioxidant and antimicrobial agents and molecular docking studies. J. Mol. Struct..

[12] Saeedi M., Mohammadi-Khanaposhtani M., Pourrabia P., Razzaghi N., Ghadimi R., Imanparast S., Rastegar H. (2019). Design and synthesis of novel quinazolinone-1,2,3-triazole hybrids as new anti-diabetic agents: In vitro α-glucosidase inhibition, kinetic, and docking study. Bioorg. Chem..

[13] Gholampour M., Ranjbar S., Edraki N., Mohabbati M., Firuzi O., Khosh-neviszadeh M. (2019). Click chemistry-assisted synthesis of novel aminonaphtho- quinone-1,2,3-triazole hybrids and investigation of their cytotoxicity and cancer cell cycle alterations. Bioorg. Chem..

[14] Chinthala Y., Thakur S., Tirunagari S., Chinde S., Domatti A.K., Arigari N.K., Tiwari A. (2015). Synthesis, docking and ADMET studies of novel chalcone triazoles for anti-cancer and anti-diabetic activity. Eur. J. Med. Chem..

[15] Rao P.S., Kurumurthy C., Veeraswamy B., Kumar G.S., Poornachandra Y., mar C.G.K., Vasamsetti S.B., Kotamraju S., Narsaiah B. (2014). Synthesis of novel 1,2,3-triazole substituted- N -alkyl/aryl nitrone derivatives, their anti-inflammatory and anti-cancer activity. Eur. J. Med. Chem..

[16] Alminderej F.M., Elganzory H.H., El-Bayaa M.N., Awad H.M., El-Sayed W.A. (2019). Synthesis and cytotoxic activity of new 1, 3, 4-thiadiazole thioglycosides and 1, 2, 3-triazolyl-1, 3, 4-thiadiazole N-glycosides. Molecules.

[17] Gür M., Yerlikaya S., Şener N., Özkınalı S., Baloglu M.C., Gökçe H., Altunoglu Y.C., Demir S., Şener İ. (2020). Antiproliferative-antimicrobial properties and structural analysis of newly synthesized Schiff bases derived from some 1, 3, 4-thiadiazole compounds. J. Mol. Struct..

[18] El-Naggar M., Sallam H.A., Shaban S.S., Abdel-Wahab S.S., EAmr A.E.G., Azab M.E., Nossier E.S., Al-Omar M.A. (2019). Design, synthesis, and molecular docking study of novel heterocycles incorporating 1, 3, 4-thiadiazole moiety as potential antimicrobial and anticancer agents. Molecules.

[19] Khattab R.R., Alshamari A.K., Hassan A.A., Elganzory H.H., El-Sayed W.A., Awad H.M., Nossier E.S., Hassan N.A. (2021). Click chemistry based synthesis, cytotoxic activity and molecular docking of novel triazole-thienopyrimidine hybrid glycosides targeting EGFR. J. Enzym. Inhib. Med. Chem..

[20] Medvedev A., Buneeva O., Gnedenko O.V., Ershov P.V., Ivanov A. (2018). Isatin, an endogenous nonpeptide biofactor: A review of its molecular targets, mechanisms of actions, and their biomedical implications. BioFactors.

[21] Hu H., Wu J., Ao M., Zhou X., Li B., Cui Z., Wu T., Wang L., Xue Y., Wu Z. (2020). Design, synthesis and biological evaluation of methylenehydrazine-1-carboxamide derivatives with (5-((4-(pyridin-3-yl) pyrimidin-2-yl) amino)-1H-indole scaffold: Novel potential CDK9 inhibitors. Bioorg. Chem..

[22] Al-Warhi T., El Kerdawy A.M., Aljaeed N., Ismael O.E., Ayyad R.R., Eldehna W.M., Abdel-Aziz H.A., Al-Ansary G.H. (2020). Synthesis, biological evaluation and in silico studies of certain oxindole–indole conjugates as anticancer CDK inhibitors. Molecules.

[23] Mohamed A.M., Al-Qalawi H.R., El-Sayed W.A., Arafa W.A., Alhumaimess M.S., Hassan A.K. (2015). Anticancer activity of newly synthesized triazolopyrimidine derivatives and their nucleoside analogs. Acta Pol. Pharm. Drug Res..

[24] Rahman A.A., Nassar I.F., Shaban A.K., El-Kady D.S., Awad H.M., El Sayed W.A. (2019). Synthesis, docking studies into CDK-2 and anticancer activity of new derivatives based pyrimidine scaffold and their derived glycosides. Mini. Rev. Med. Chem..

[25] Halay E., Ay E., Şalva E., Ay K., Karayıldırım T. (2017). Syntheses of 1,2,3-triazolebridged pyranose sugars with purine and pyrimidine nucleobases and evaluation of their anticancer potential. Nucleosides Nucleotides Nucleic Acids.

[26] Ghorab M.M., Alsaid M.S., Soliman A.M. (2018). Dual EGFR/HER2 inhibitors and apoptosis inducers: New benzo [g] quinazoline derivatives bearing benzenesulfonamide as anticancer and radiosensitizers. Bioorg. Chem..

[27] Das D., Xie L., Wang J., Xu X., Zhang Z., Shi J., Le X., Hong J. (2019). Discovery of new quinazoline derivatives as irreversible dual EGFR/HER2 inhibitors and their anticancer activities–Part 1. Bioorganic Med. Chem. Lett..

[28] Ahmed S., Sami A., Xiang J. (2015). HER2-directed therapy: Current treatment options for HER2-positive breast cancer. Breast Cancer.

[29] Ross J.S., Slodkowska E.A., Symmans W.F., Pusztai L., Ravdin P.M., Hortobagyi G.N. (2009). The HER-2 receptor and breast cancer: Ten years of targeted anti–HER-2 therapy and personalized medicine. Oncologist.

[30] Eroglu Z., Tagawa T., Somlo G. (2014). Human epidermal growth factor receptor familytargeted therapies in the treatment of HER2-overexpressing breast cancer. Oncologist.

[31] Hsu J.L., Hung M.-C. (2016). The role of HER2, EGFR, and other receptor tyrosine kinases in breast cancer. Cancer Metastasis Rev..

[32] Elferink L.A., Resto V.A. (2011). Receptor-Tyrosine-Kinase-Targeted Therapies for Head and Neck Cancer. J. Signal Transduct..

[33] Schuler M., Yang J.H., Park K., Kim J.H., Bennouna J., Chen Y.M., Chouaid C., De Marinis F., Feng J.F., Grossi F. (2016). Afatinib beyond progression in patients with non-small-cell lung cancer following chemotherapy, erlotinib/gefitinib and afatinib: Phase III randomized LUX-Lung 5 trial. Ann. Oncol..

[34] El-Sharief A.M.S., Ammar Y.A., Belal A., El-Sharief M.A.S., Mohamed Y.A., Mehany A.B., Ali G.A.E., Ragab A. (2019). Design, synthesis, molecular docking and biological activity evaluation of some novel indole derivatives as potent anticancer active agents and apoptosis inducers. Bioorg. Chem..

[35] Nagarsenkar A., Prajapti S.K., Guggilapu S.D., Birineni S., Kotapalli S.S., Ummanni R., Babu B.N. (2016). Investigation of triazole-linked indole and oxindole glycoconjugates as potential anticancer agents: Novel Akt/PKB signaling pathway inhibitors. Med. Chem. Comm..

[36] Abbas H.S., el Sayed W.A., Fathy N.M. (2010). Synthesis and antitumor activity of new dihydropyridine thioglycosides and their corresponding dehydrogenated forms. Eur. J. Med. Chem..

[37] Nassar I.F., el Kady D.S., Awad H.M., El-Sayed W.A. (2019). Design, Synthesis, and Anticancer Activity of New Oxadiazolyl-Linked and Thiazolyl-Linked Benzimidazole Arylidines, Thioglycoside, and Acyclic Analogs. J. Heterocycl. Chem..

[38] Kassem A.F., Abbas E.M.H., El-Kady D.S., Awad H.M., El-Sayed W.A. (2019). Design, synthesis and anticancer activity of new thiazole-tetrazole or triazole hybrid glycosides targeting CDK-2 via structure-based virtual screening. Mini Rev. Med. Chem..

[39] Yousif M.N.M., El-Sayed W.A., Abbas H.A.S., Awad H.M., Yousif N.M. (2019). Anticancer activity of new substituted pyrimidines, their thioglycosides and thiazolopyrimidine derivatives. J. Appl. Pharm. Sci..

[40] Flefel E.E., Tantawy W.A., El-Sayed W.A., Sayed H.H., Fathy N.M. (2013). Synthesis and Anticancer Activity of New Substituted Pyrazoles and Their Derived 1,2,4-Triazoles and Their Sugar Derivatives. J. Hetererocyclic Chem..

[41] Abdel-Aal M.T., El-Sayed W.A., AH A.A., El Ashry E.S. (2003). Synthesis of some functionalized arylaminomethyl-1, 2, 4-triazoles, 1, 3, 4-oxa-and thiadiazoles. Die Pharmazie.

[42] Srour A.M., El-Bayaa M.N., Omran M.M., Sharaky M.M., El-Sayed W.A. (2021). Synthesis and Cytotoxic Properties of New Substituted Glycosides-Indole Conjugates as Apoptosis Inducers in Cancer Cells. Anti-Cancer Agents Med. Chem..

[43] Hassan A.S., Moustafa G.O., Awad H.M., Nossier E.S., Mady M.F. (2021). Design, Synthesis, Anticancer Evaluation, Enzymatic Assays, and a Molecular Modeling Study of Novel Pyrazole–Indole Hybrids. ACS Omega.

[44] Abd El-Meguid E.A., Moustafa G.O., Awad H.M., Zaki E.R., Nossier E.S. (2021). Novel benzothiazole hybrids targeting EGFR: Design, synthesis, biological evaluation and molecular docking studies. J. Mol. Struct..

[45] Othman I.M., Gad-Elkareem M.A., Amr A.E.G.E., Al-Omar M.A., Nossier E.S., Elsayed E.A. (2020). Novel heterocyclic hybrids of pyrazole targeting dihydrofolate reductase: Design, biological evaluation and in silico studies. J. Enzym. Inhib. Med. Chem..

[46] Hashem H.E., Amr A.E.G.E., Nossier E.S., Elsayed E.A., Azmy E.M. (2020). Synthesis, antimicrobial activity and molecular docking of novel thiourea derivatives tagged with thiadiazole, imidazole and triazine moieties as potential DNA gyrase and topoisomerase IV inhibitors. Molecules.

[47] Khattab R.R., Hassan A.A., AOsman D.A., Abdel-Megeid F.M., Awad H.M., Nossier E.S., El-Sayed W.A. (2021). Synthesis, anticancer activity and molecular docking of new triazolo [4, 5-d] pyrimidines based thienopyrimidine system and their derived N-glycosides and thioglycosides. Nucleosides Nucleotides Nucleic Acids.

[48] Yoshikawa S., Kukimoto-Niino M., Parker L., Handa N., Terada T., Fujimoto T., Terazawa Y., Wakiyama M., Sato M., Sano S. (2013). Structural basis for the altered drug sensitivities of non-small cell lung cancer-associated mutants of human epidermal growth factor receptor. Oncogene.

[49] Ishikawa T., Seto M., Banno H., Kawakita Y., Oorui M., Taniguchi T., Ohta Y., Tamura T., Nakayama A., Miki H. (2011). Design and synthesis of novel human epidermal growth factor receptor 2 (HER2)/epidermal growth factor receptor (EGFR) dual inhibitors bearing a pyrrolo [3, 2-d] pyrimidine scaffold. J. Med. Chem..

[50] Abuelizz H.A., Marzouk M., Bakheit A.H., Awad H.M., Soltan M.M., Naglah A.M., Al-Salahi R. (2020). Antiproliferative and Antiangiogenic Properties of New VEGFR-2-targeting 2-thioxobenzo [g] quinazoline Derivatives (In Vitro). Molecules.

[51] Nossier E.S., El-Hallouty S.M., Zaki E.R. (2015). Synthesis, anticancer evaluation and molecular modeling of some substituted thiazolidinonyl and thiazolyl pyrazole derivatives. Int. J. Pharm. Sci..

[52] Dawood D.H., Nossier E.S., Ali M.M., Mahmoud A.E. (2020). Synthesis and molecular docking study of new pyrazole derivatives as potent anti-breast cancer agents targeting VEGFR-2 kinase. Bioorg. Chem..

[53] Othman I.M., Alamshany Z.M., Tashkandi N.Y., Gad-Elkareem M.A., Anwar M.M., Nossier E.S. (2021). New pyrimidine and pyrazole-based compounds as potential EGFR inhibitors: Synthesis, anticancer, antimicrobial evaluation and computational studies. Bioorg. Chem..

