# Supplementary material for: Design, Synthesis, Anticancer Activity and Molecular Docking of New 1,2,3-Triazole-Based Glycosides Bearing 1,3,4-Thiadiazolyl, Indolyl and Arylacetamide Scaffolds
Source: Molecules. 2022 Oct 17;27(20):6960. doi: 10.3390/molecules27206960 (PMC9611297; doi:10.3390/molecules27206960)

Supplementary material

Design, Synthesis, Anticancer Activity and Molecular Docking of New 1,2,3-Triazole Based Glycosides Bearing 1,3,4-Thiadiazolyl, indolyl and Arylacetamide Scaffolds

Hussein H. Elganzory [1], Fahad M. Alminderej [1,*], Mohamed N. El-Bayaa [2,*], Hanem M. Awad [3], Eman S. Nossier[4] and Wael A. El-Sayed [1,3]

[1] Department of Chemistry, College of Science, Qassim University, Buraidah 51452, Saudi Arabia
[2] Photochemistry Department, National Research Centre, Cairo 12622, Egypt
[3] Tanning Materials and Leather Technology Department, National Research Centre, El-Behouth St, Dokki, Cairo 12622, Egypt
[4] Department of Pharmaceutical Medicinal Chemistry and Drug Design, Faculty of Pharmacy (Girls), Al-Azhar University, Cairo 11754, Egypt
* Correspondence: f.alminderej@qu.edu.sa.

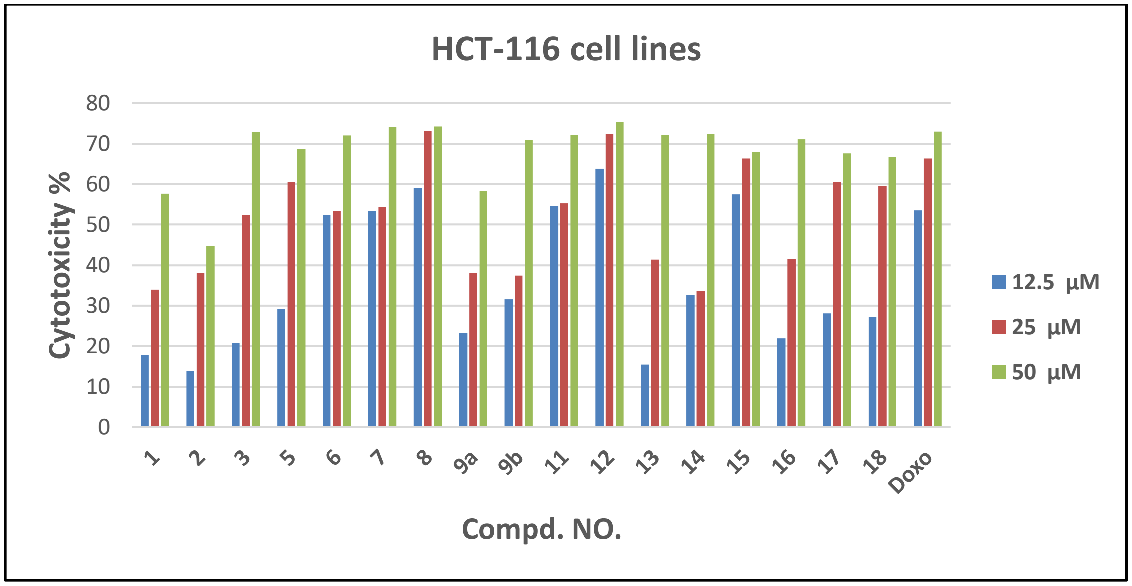

Figure S1. Dose dependent cytotoxic activity of the synthesized derivatives against HCT-116 cancer cells according to the MTT assay.

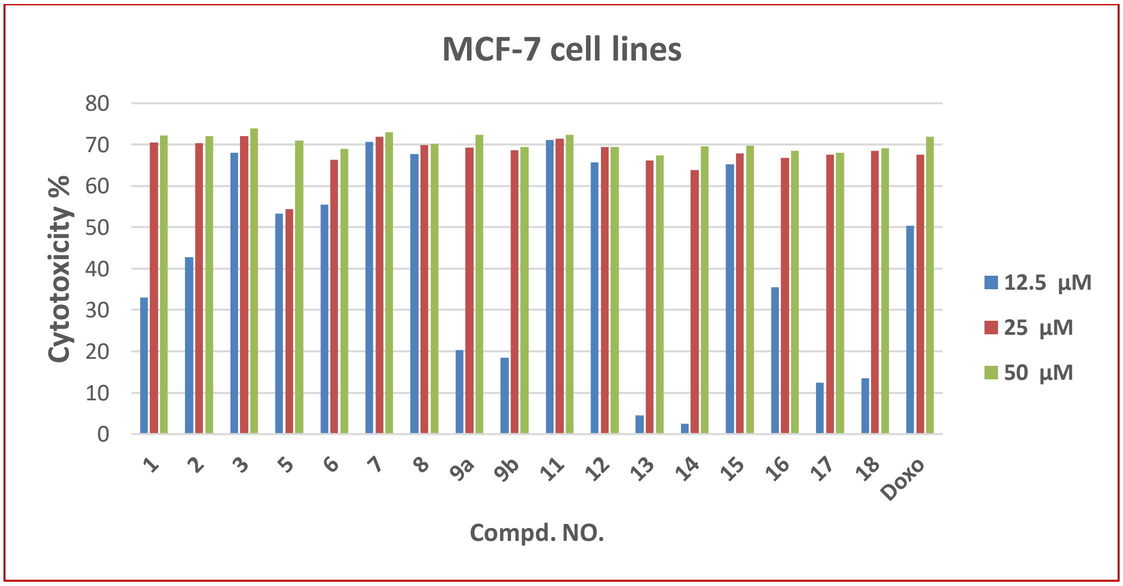

Figure S2. Dose dependent cytotoxic activity of the synthesized derivatives against MCF-7 cancer cells according to the MTT assay.

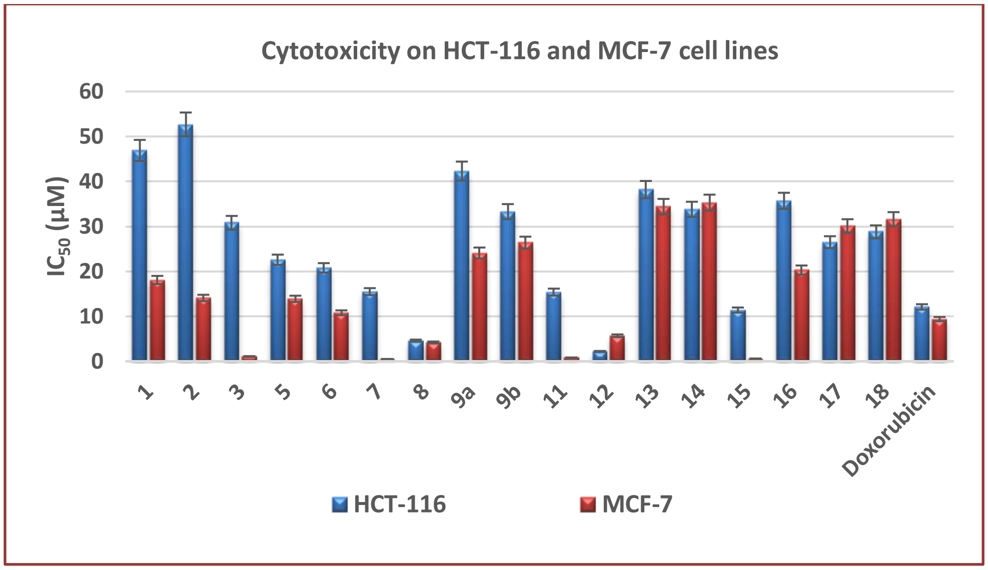

Figure S3. IC_{50} (µM) data of the screened targets in comparison with doxorubicin against human HCT-116 and MCF-7 cancer cell lines according to the MTT assay.

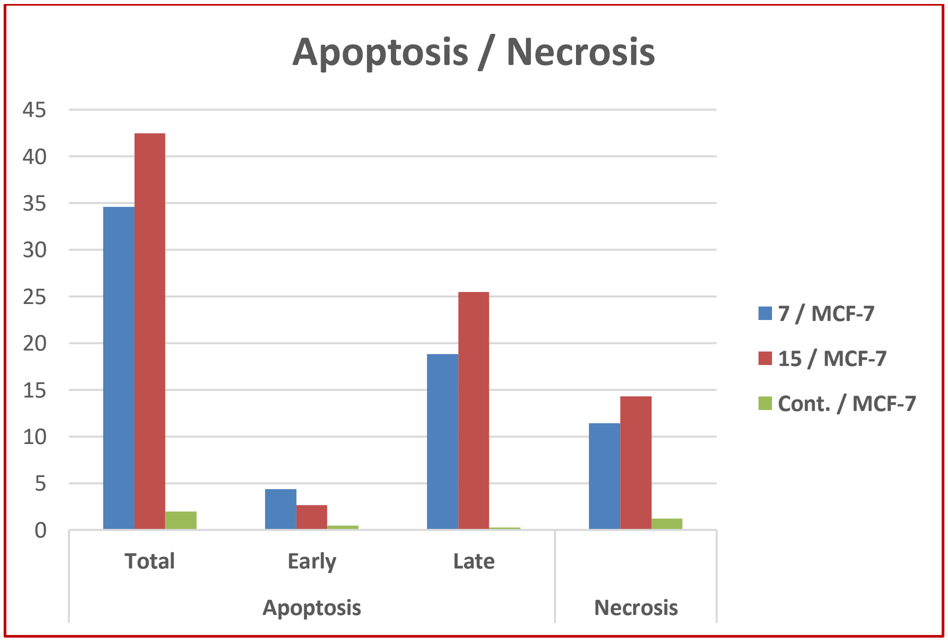

Figure S4. Apoptotic activity of the promising derivatives **7** and **15**.

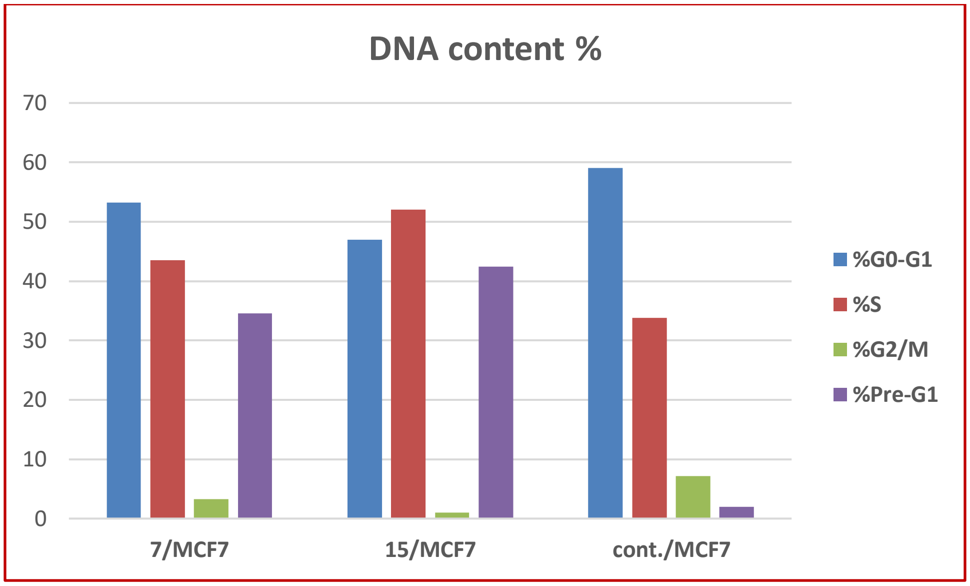

Figure S5. Cell cycle analysis in MCF-7 cells treated with the promising derivatives **7** and **15**.

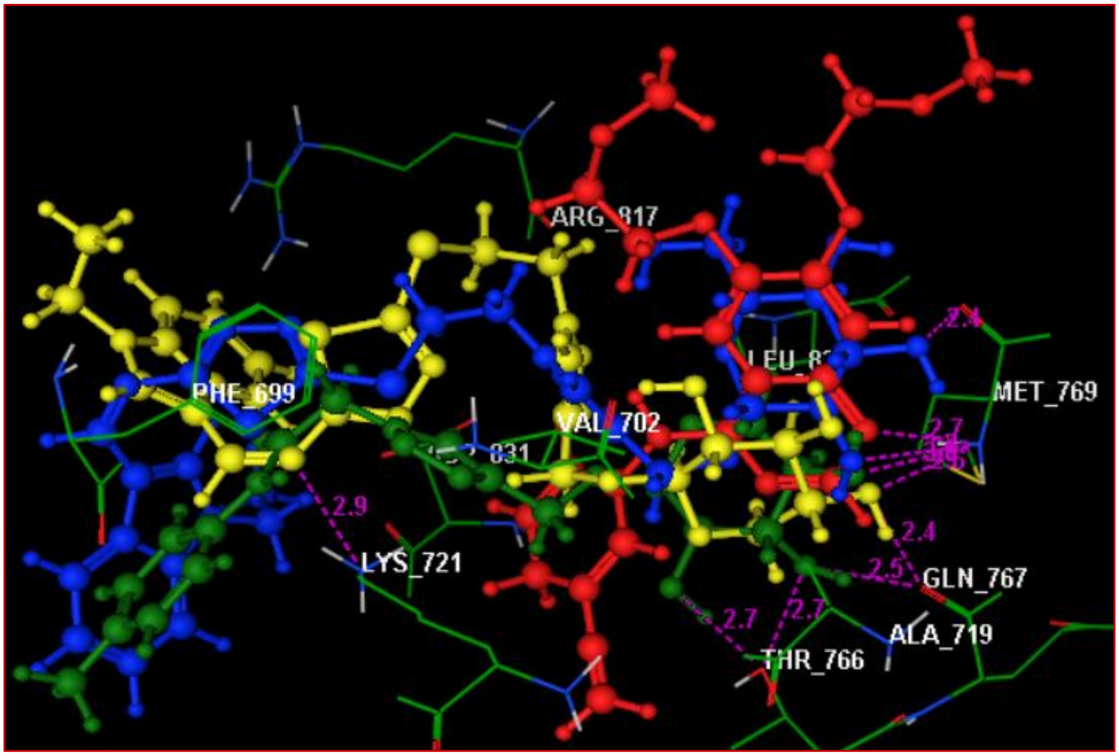

A

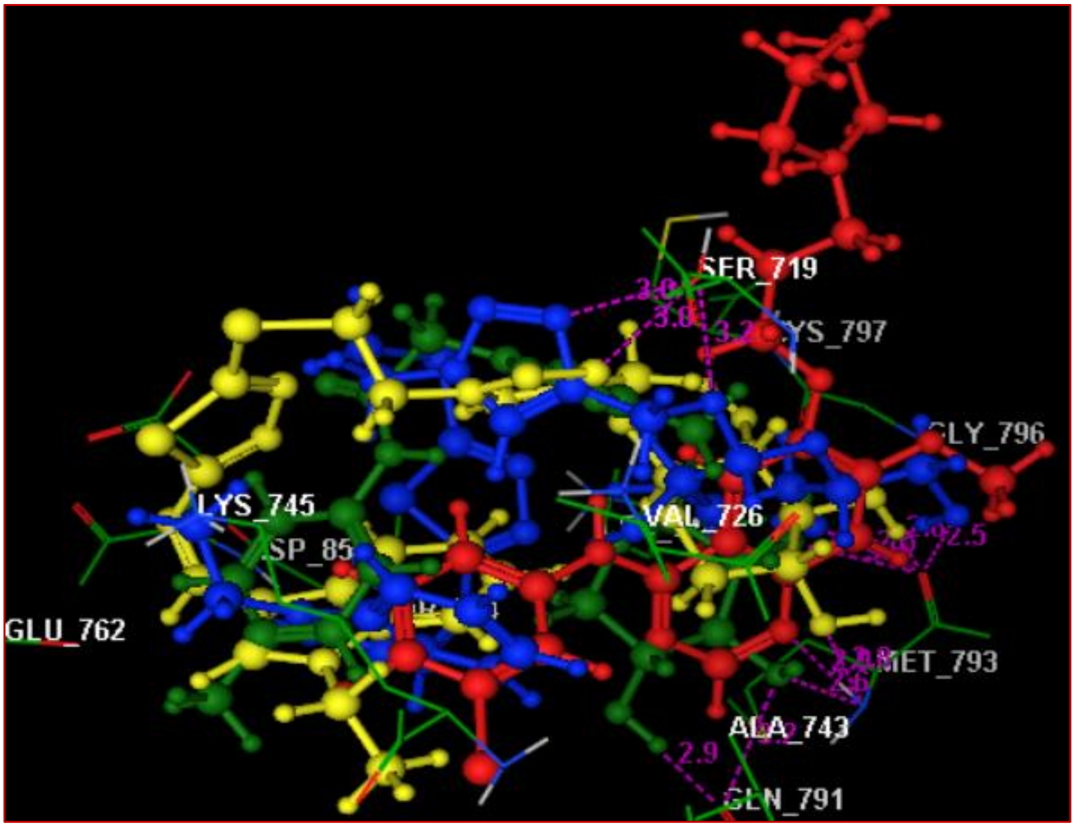

B

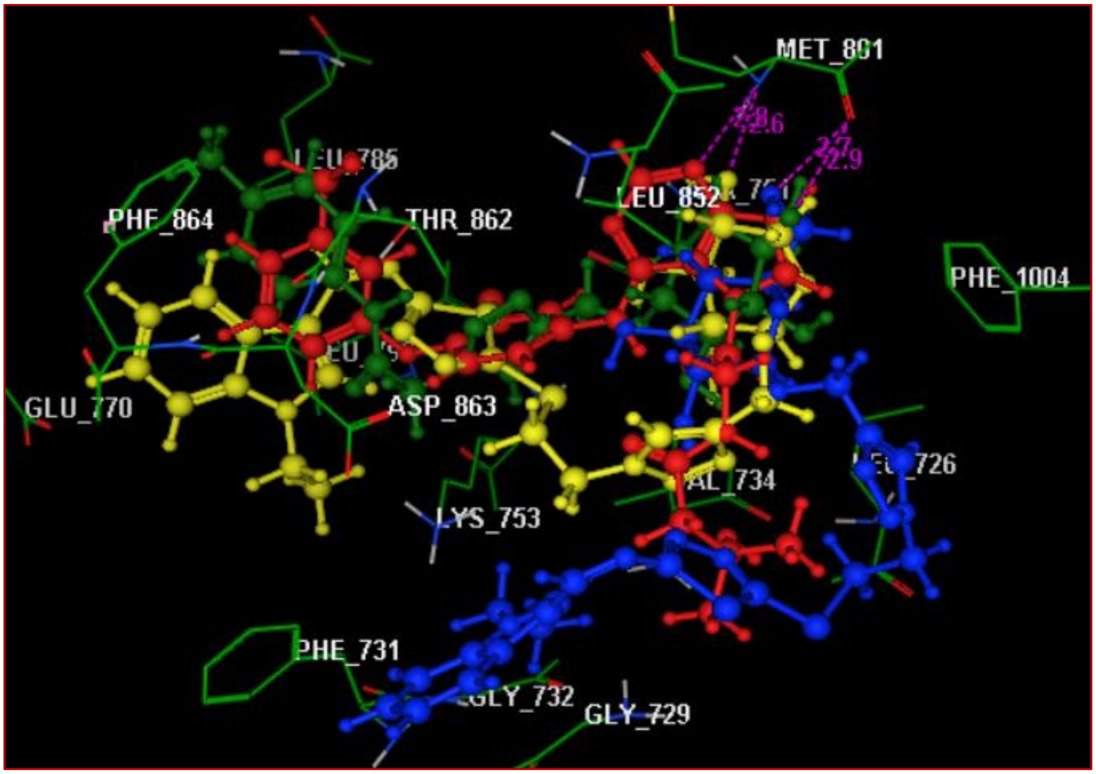

C

Figure S6. 3D diagrams of the superimposition of the original ligands erlotinib, gefitinib and TAK-285 (red), **7** (blue), **8** (yellow) and **15** (green), within the ATP-binding pockets of $EGFR^{WT}$, $EGFR^{T790M}$ and HER-2 (PDB codes: 1M17, 3UG2 and 3RCD), respectively.

^{1}H NMR for compound 2

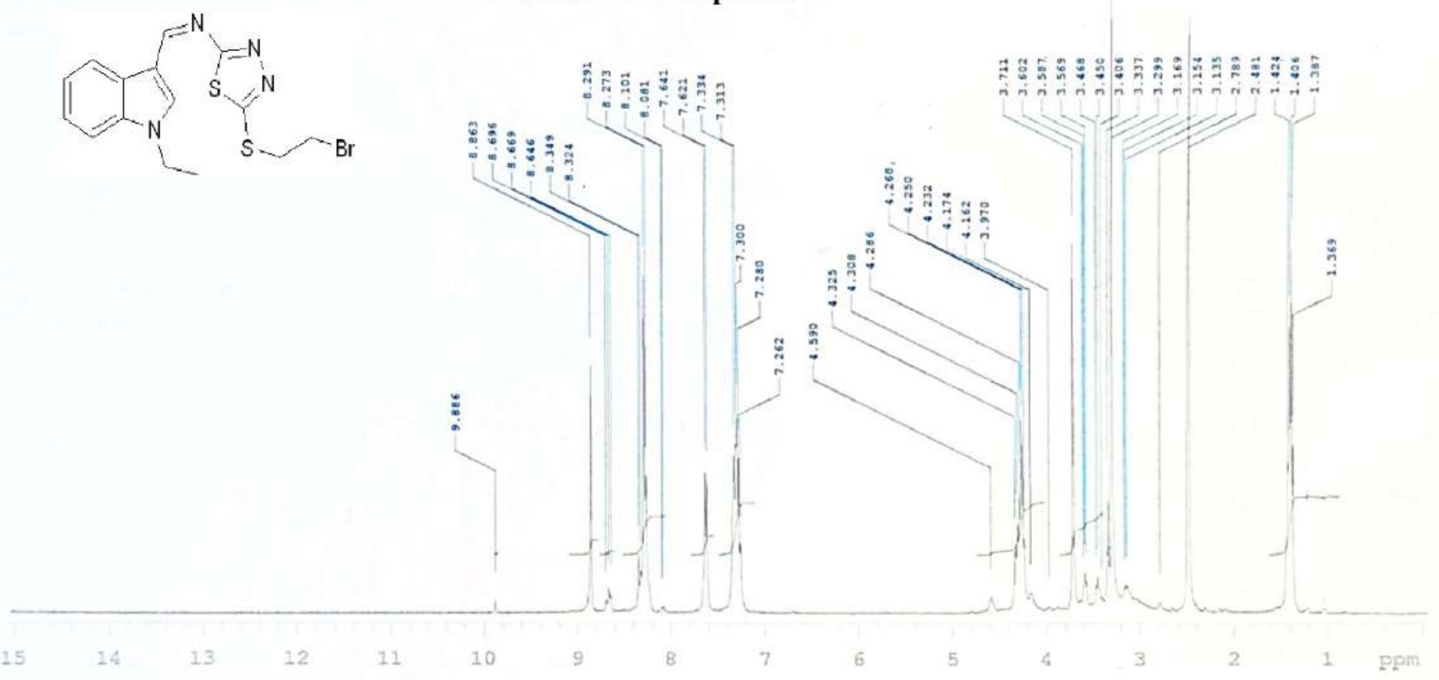

^{13}C NMR for compound 2

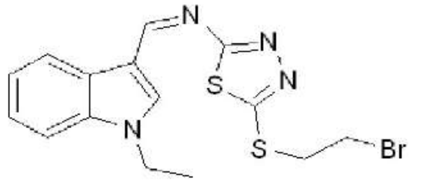

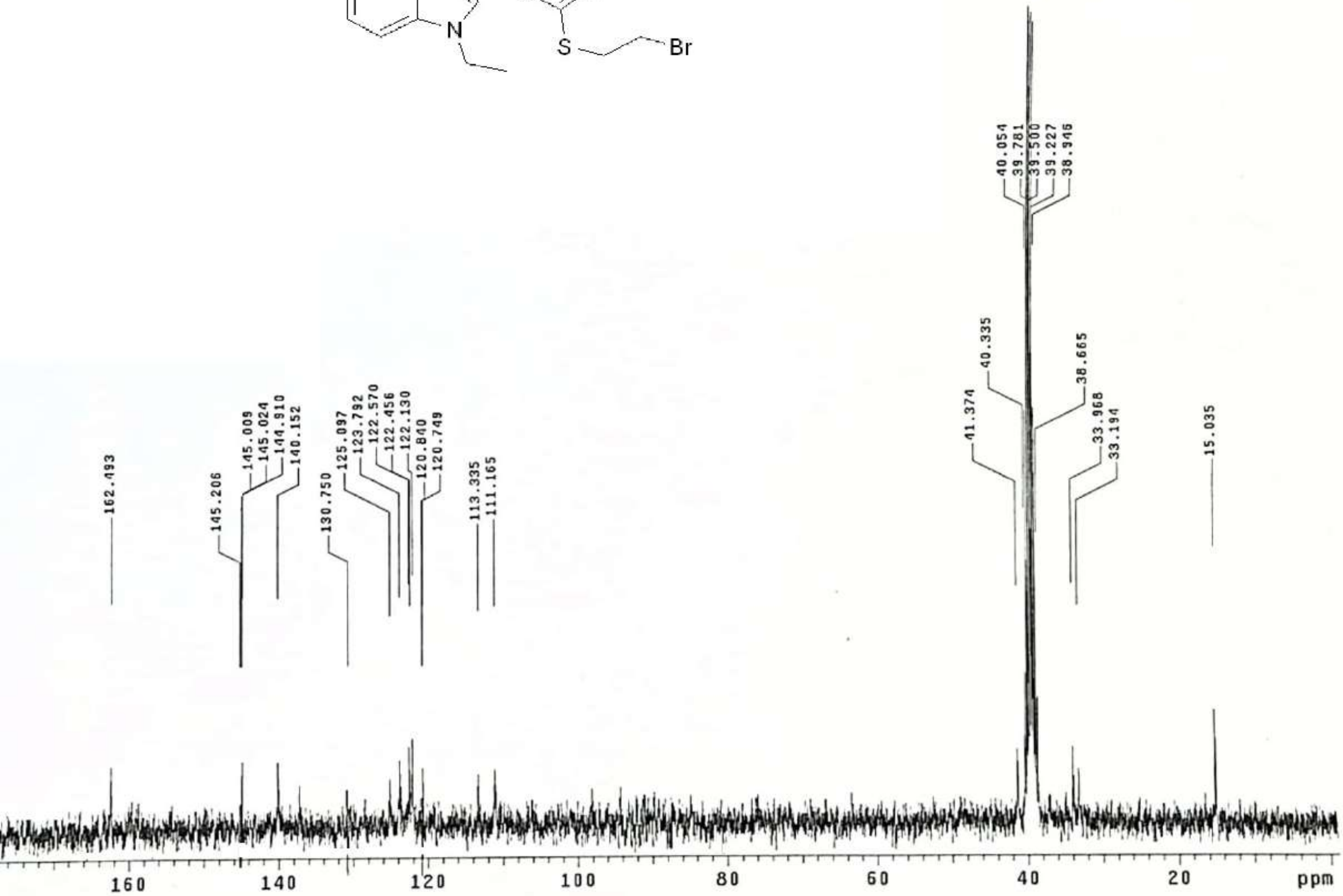

^{1}H NMR for compound 3

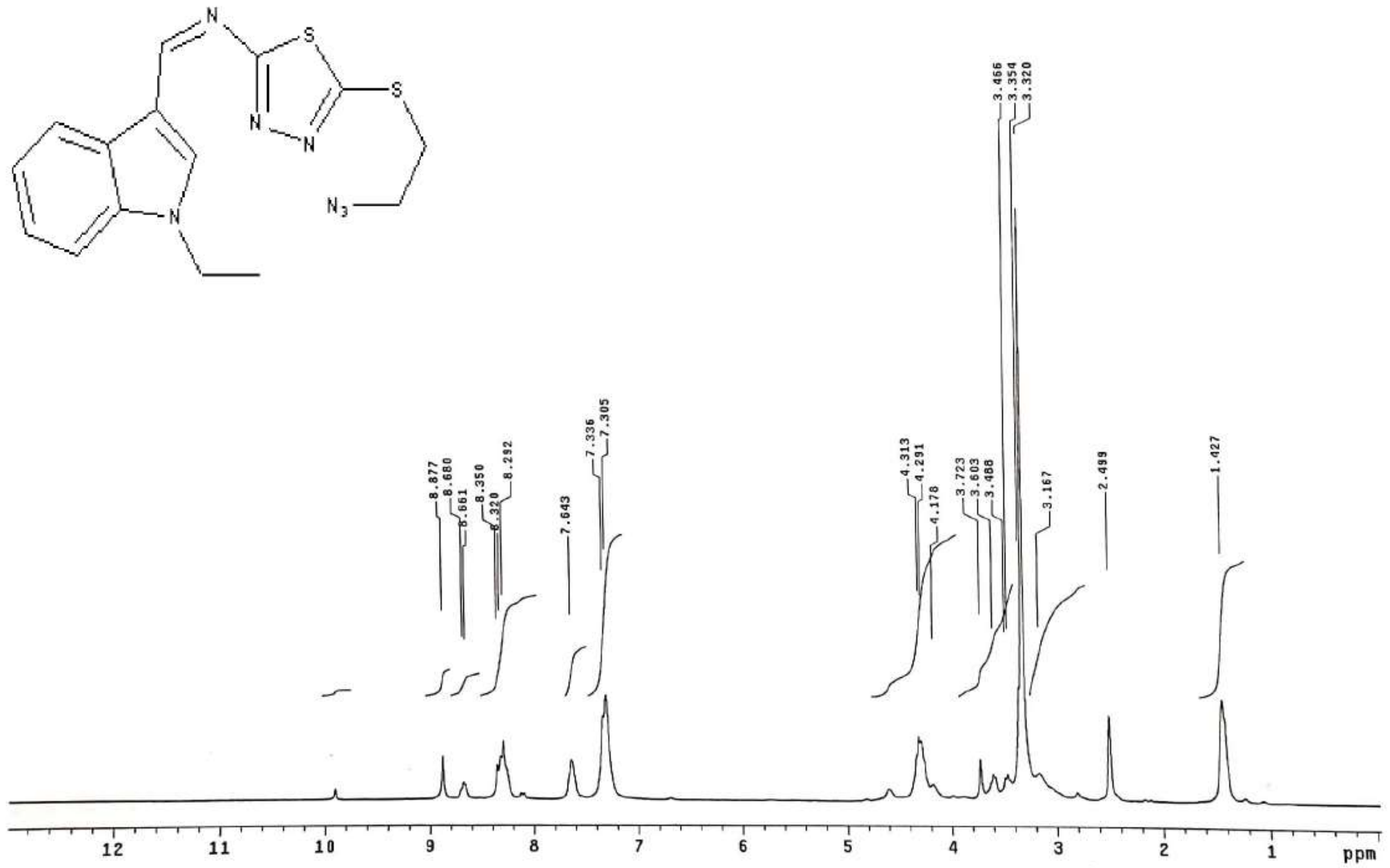

^{13}C NMR for compound 3

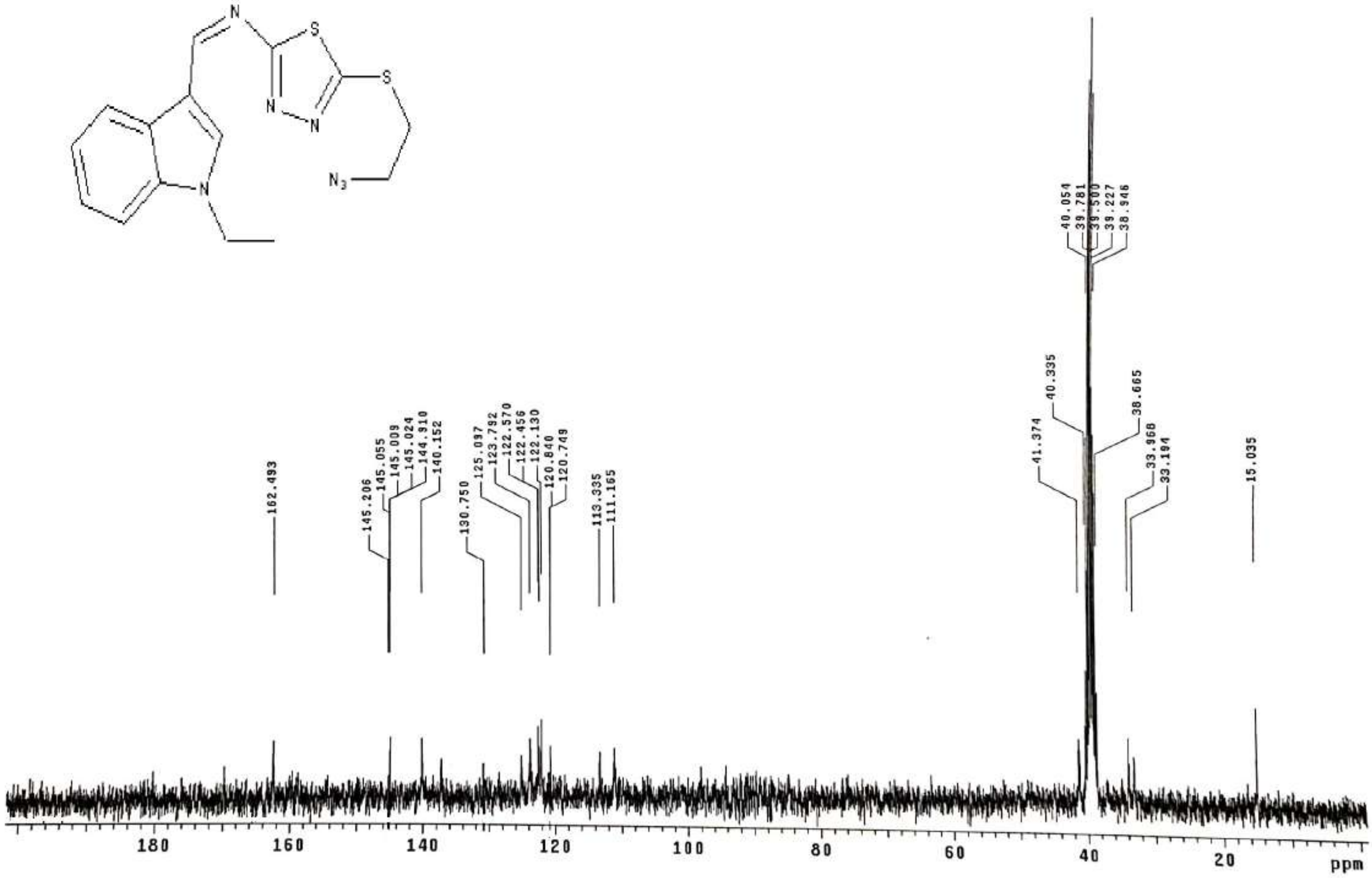

Mass for compound 3

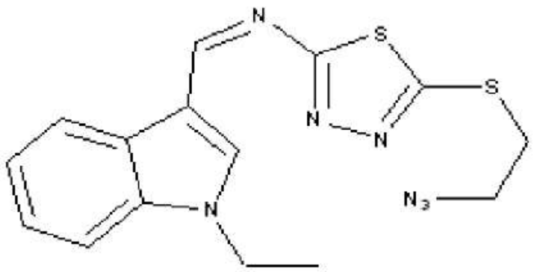

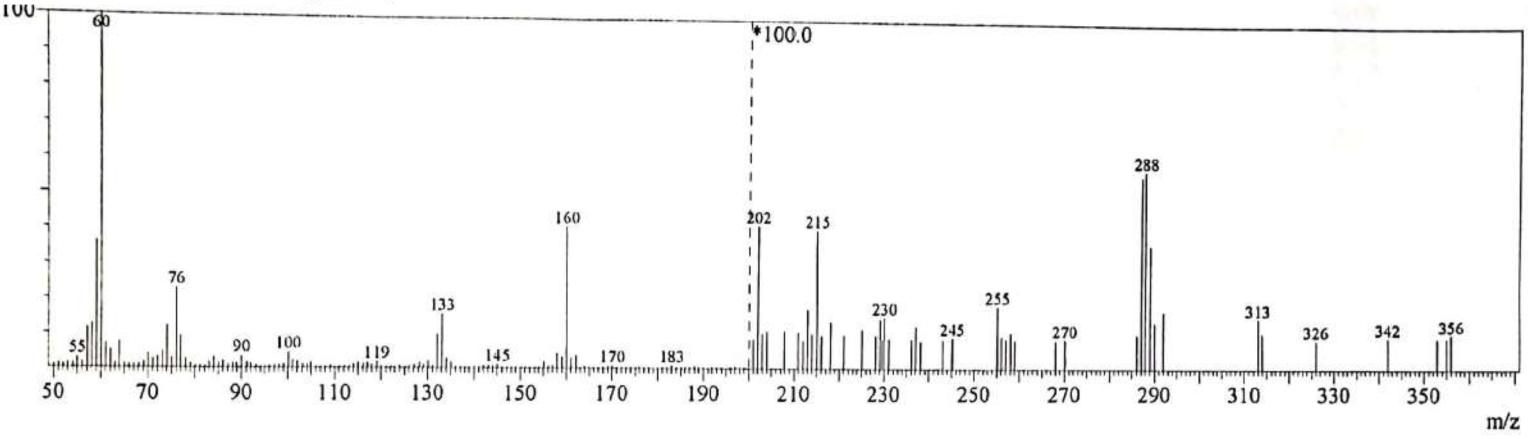

^{1}H NMR for compound 5

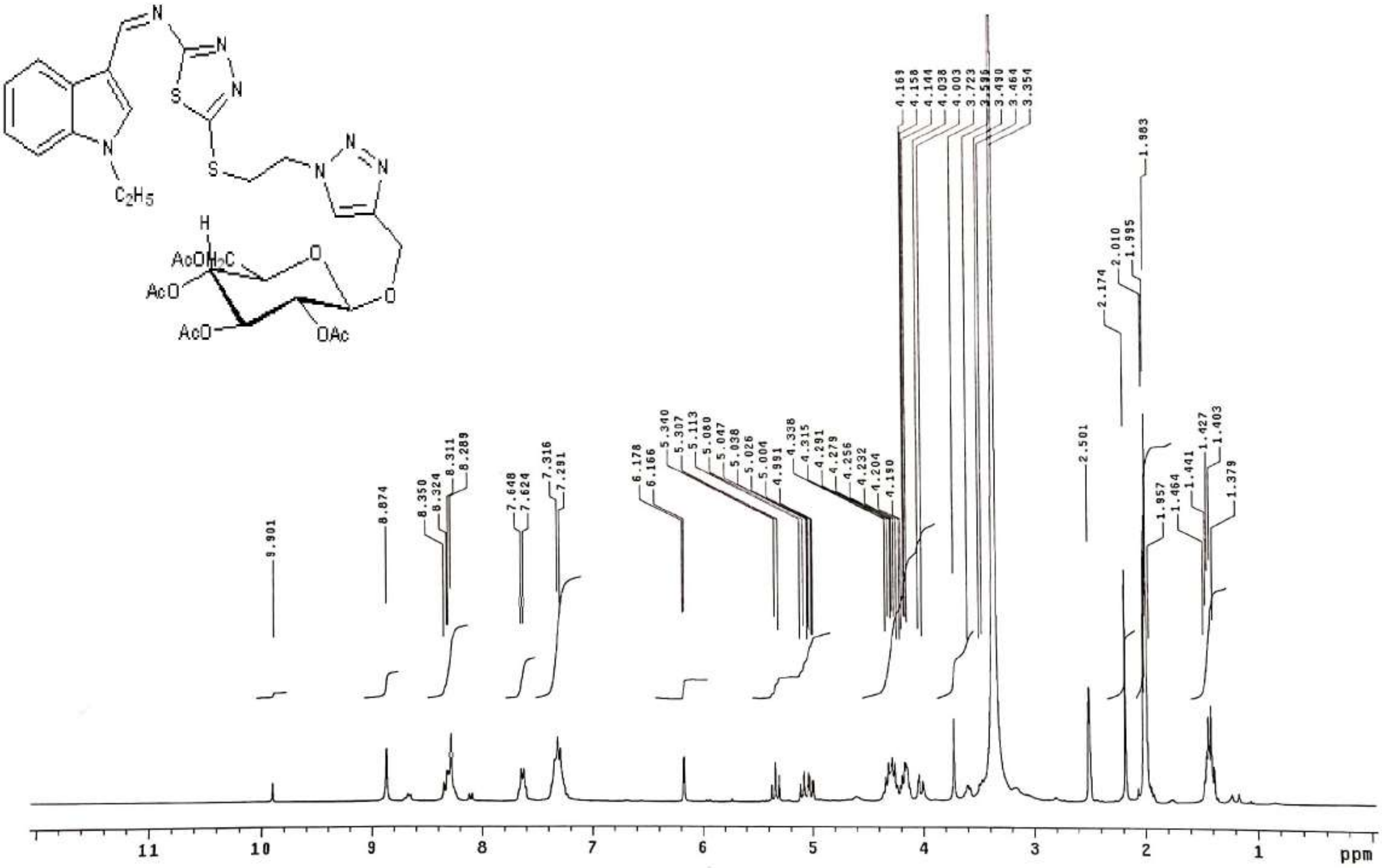

^{13}C NMR for compound 5

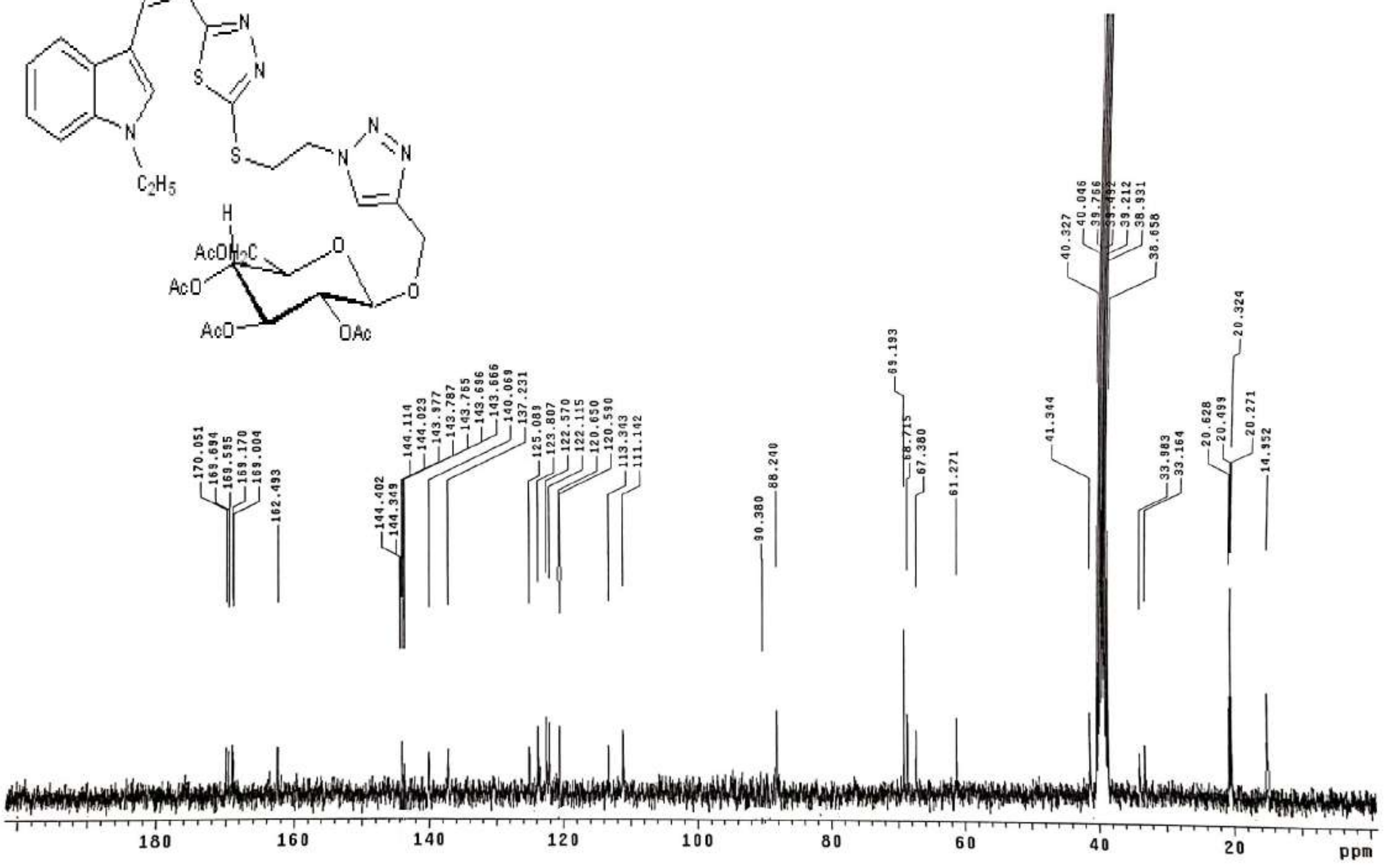

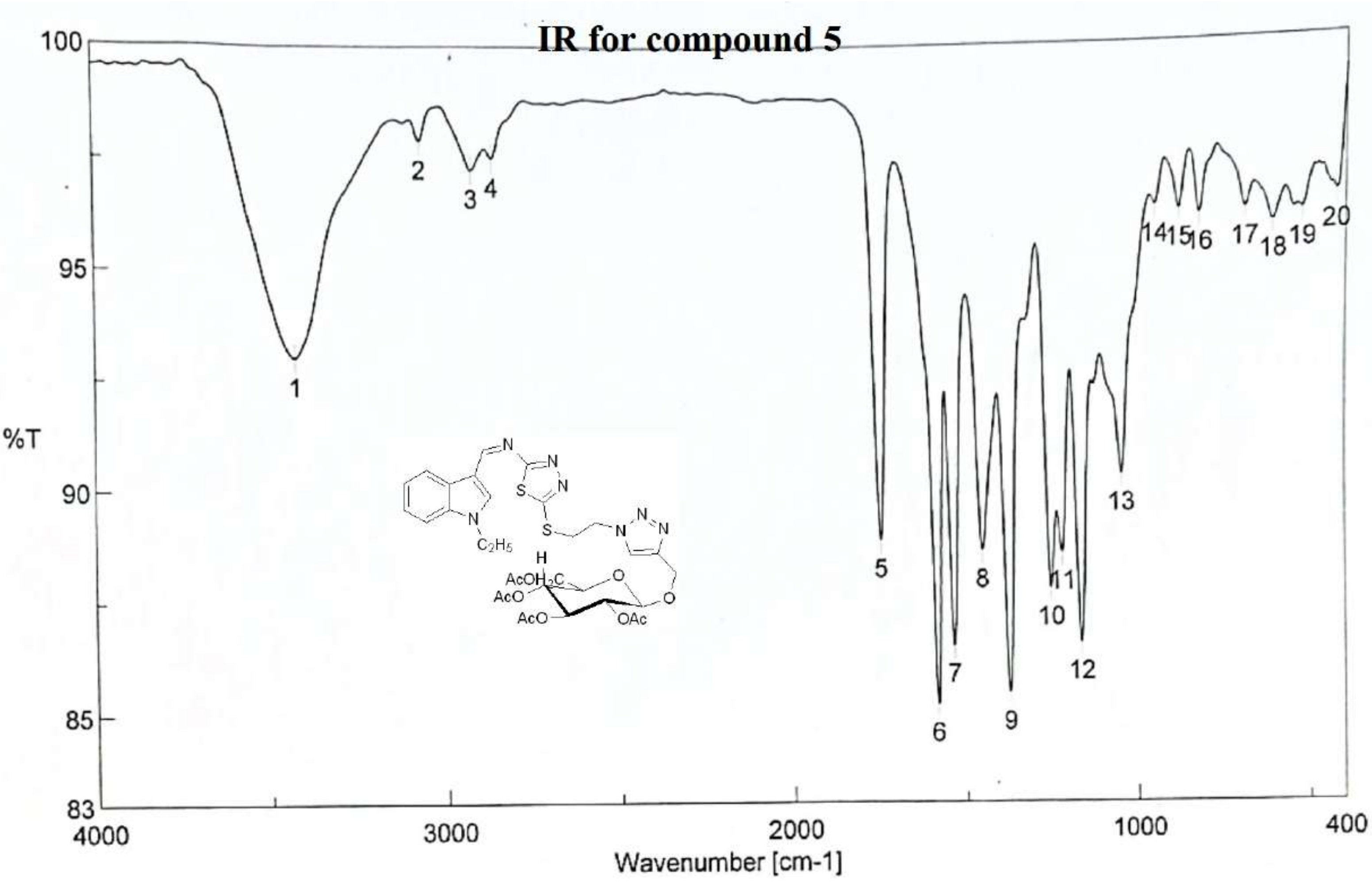
IR for compound 5
100
95
90
85
83
%T
4000
3000
2000
1000
400
Wavenumber [cm-1]
1
2
3
4
5
6
7
8
9
10
11
12
13
14
15
16
17
18
19
20
N
S
N
N
N
N
N
C2H5
H
AcOH2C
AcO
AcO
OAc
O
O

^{1}H NMR for compound 6

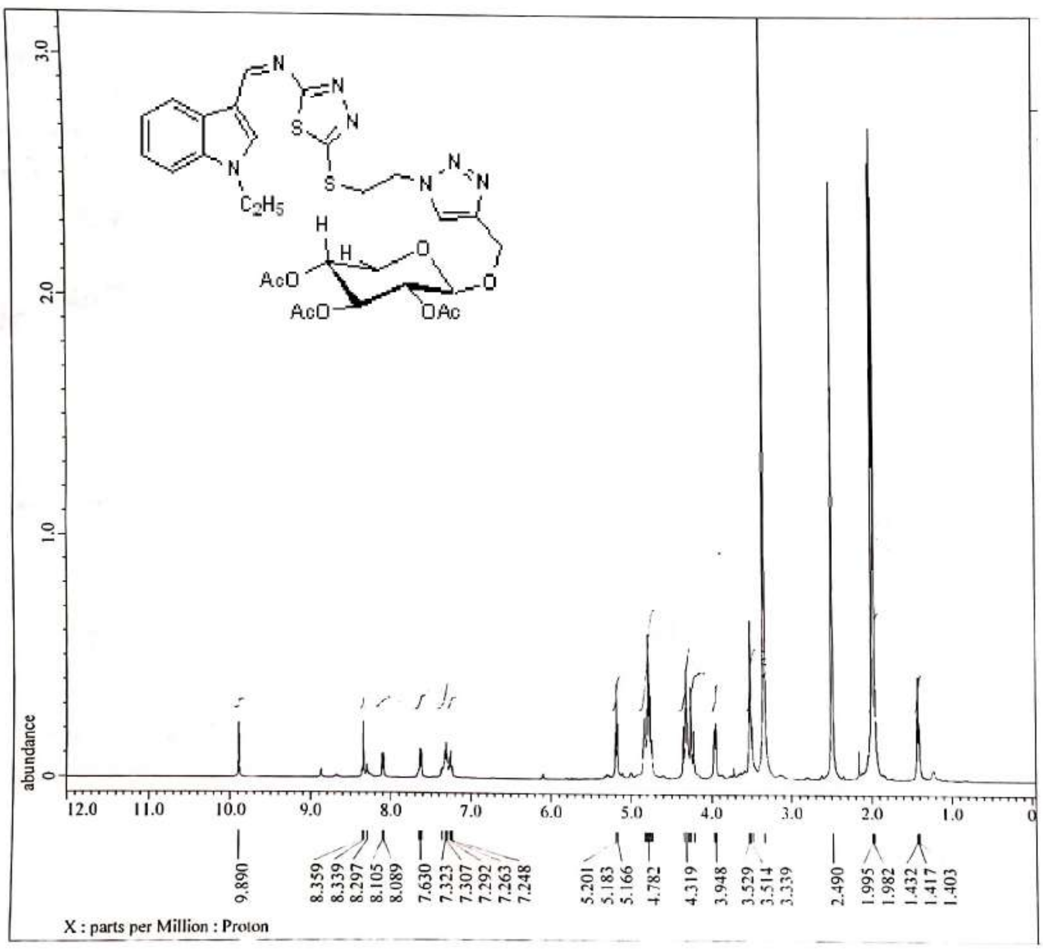

IR for compound 6

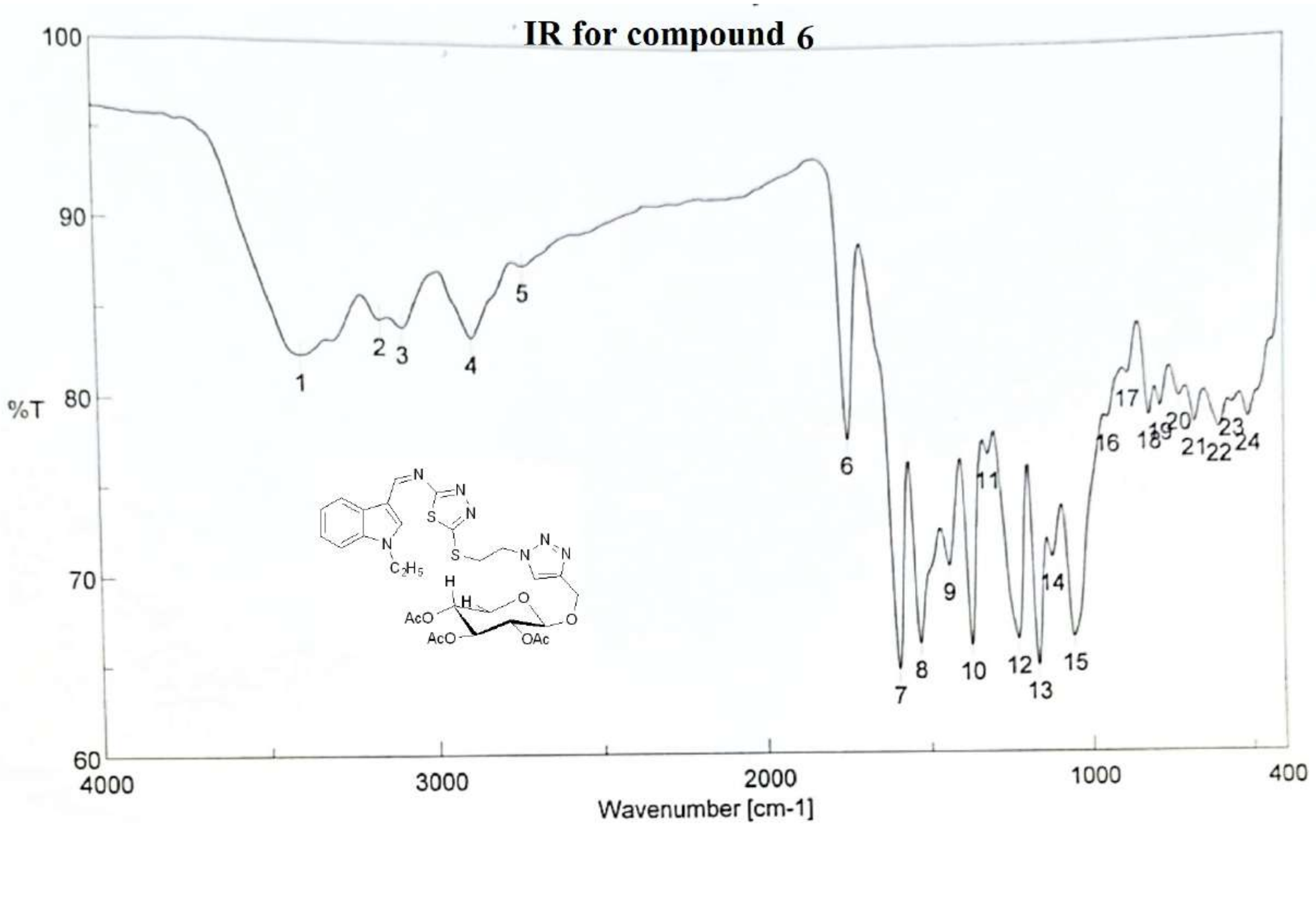

^{1}H NMR for compound 7

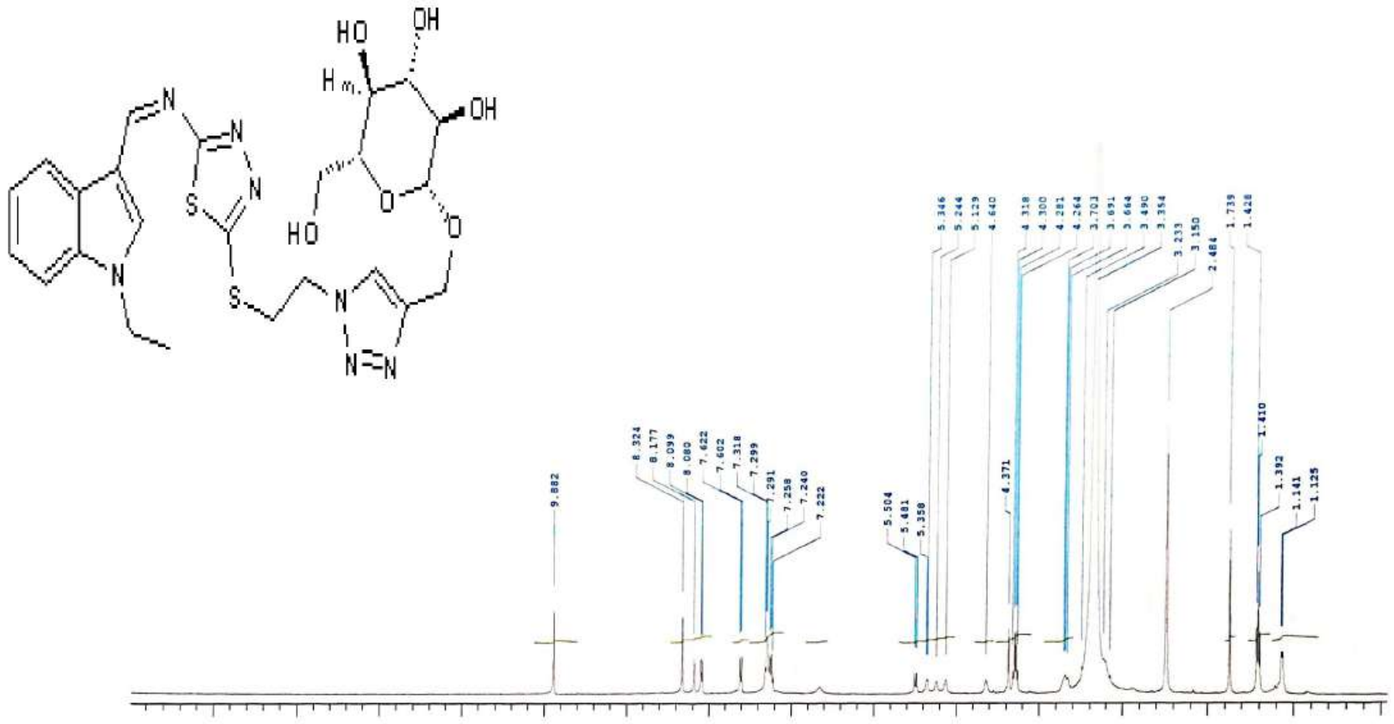

^{13}C NMR for compound 7

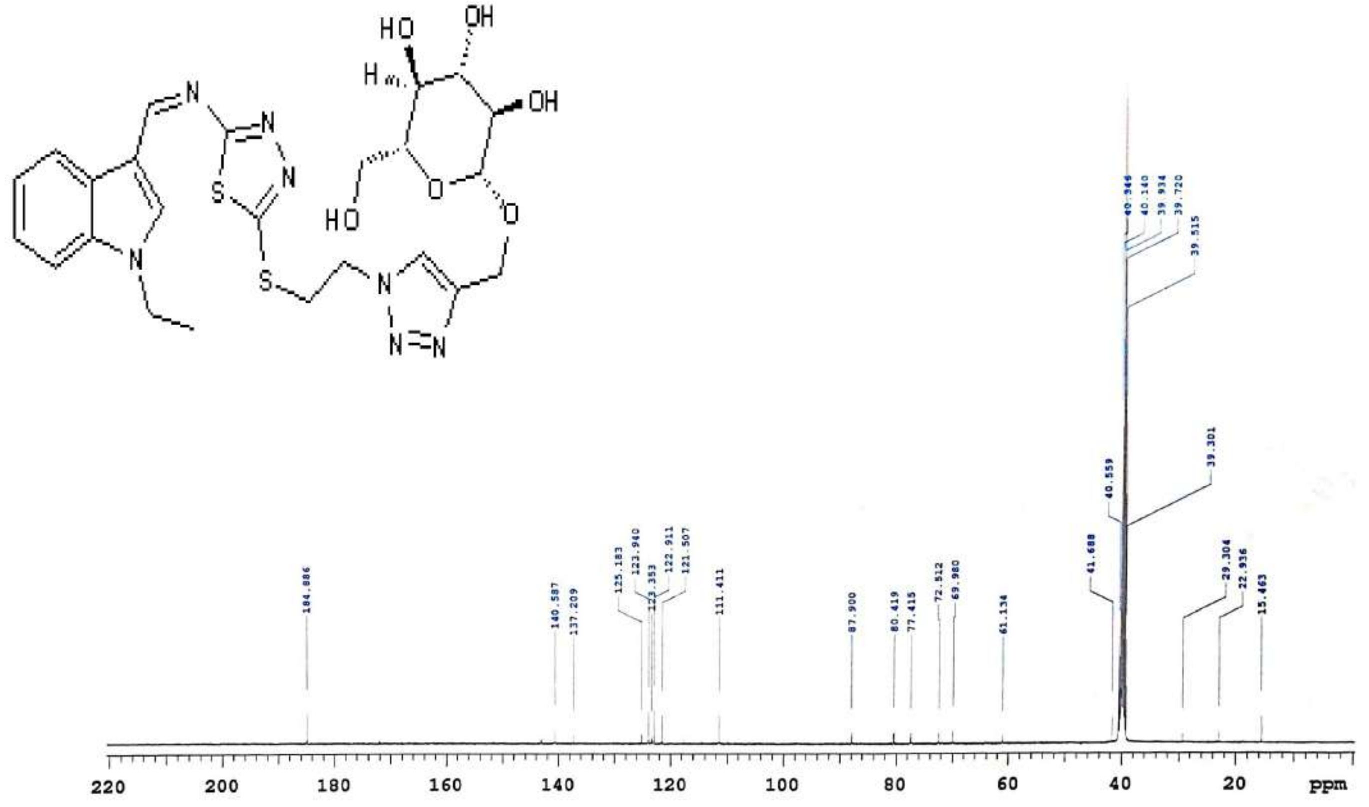

IR for compound 7

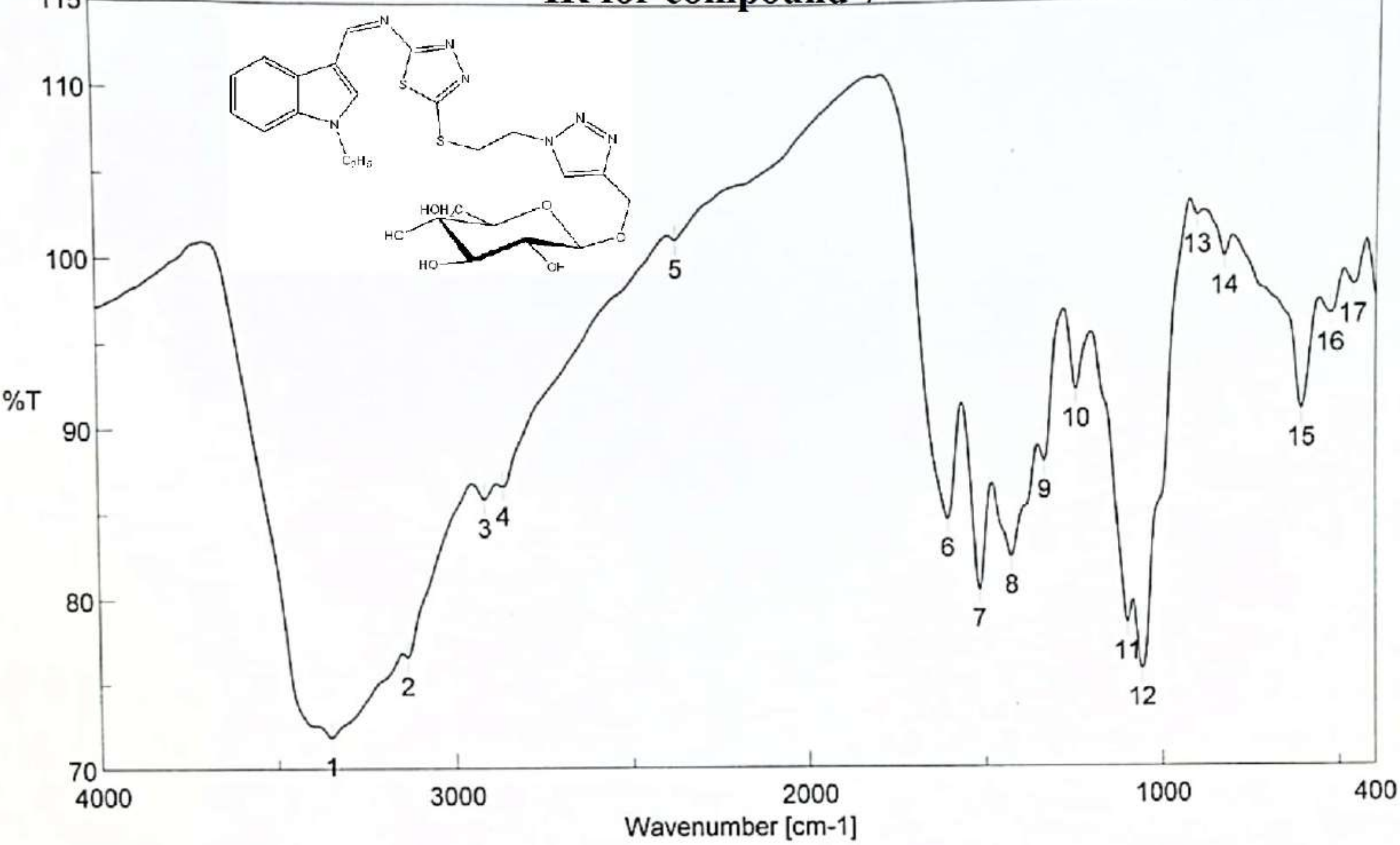

^{1}H NMR for compound 8

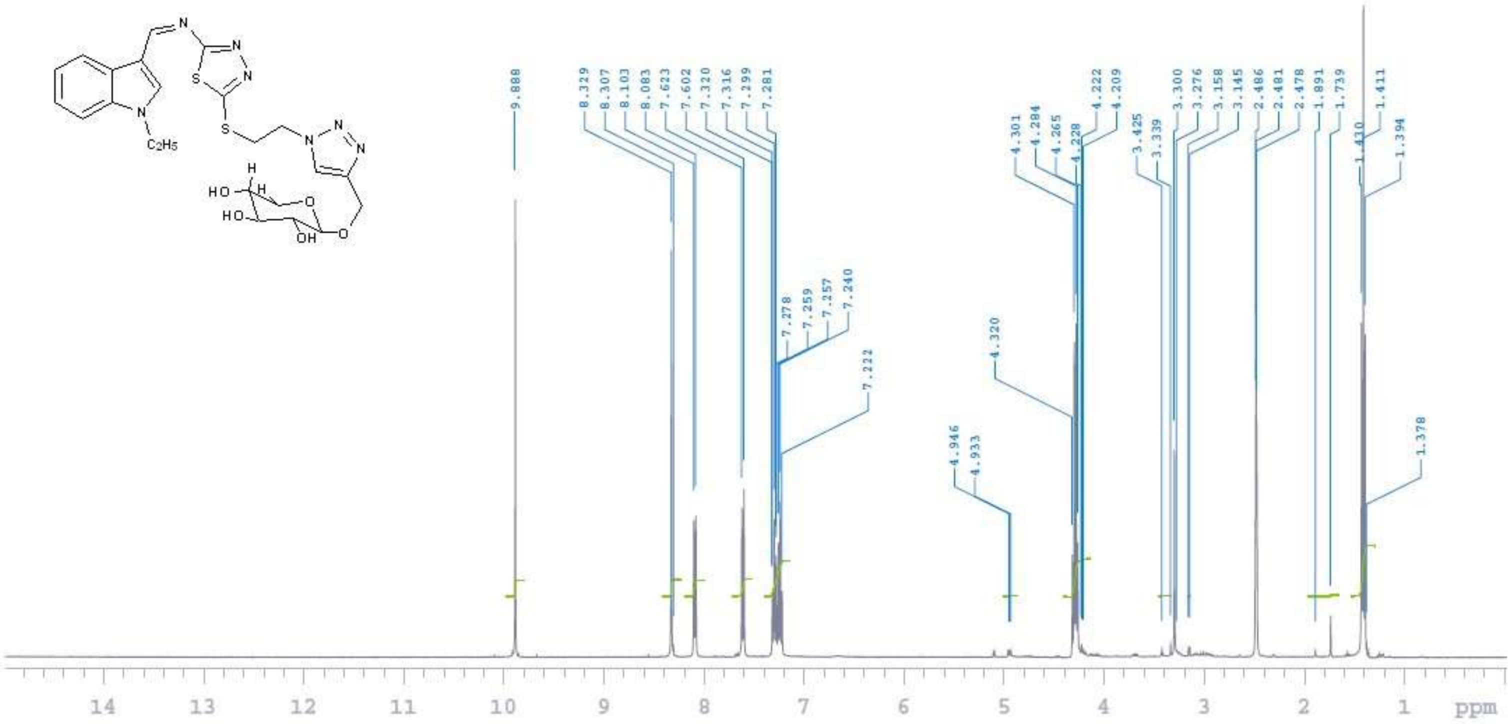

^{1}H NMR for compound 11

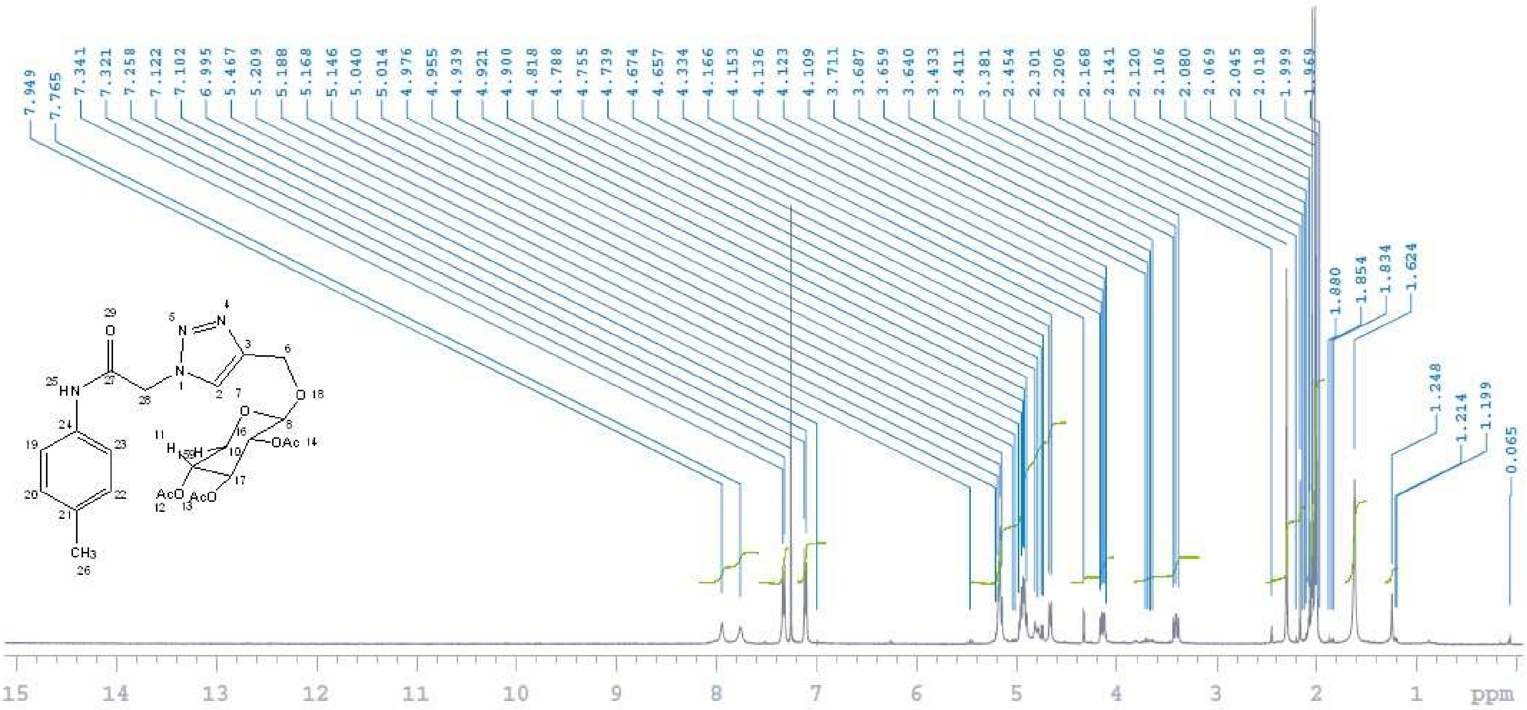

^{1}H NMR for compound 12

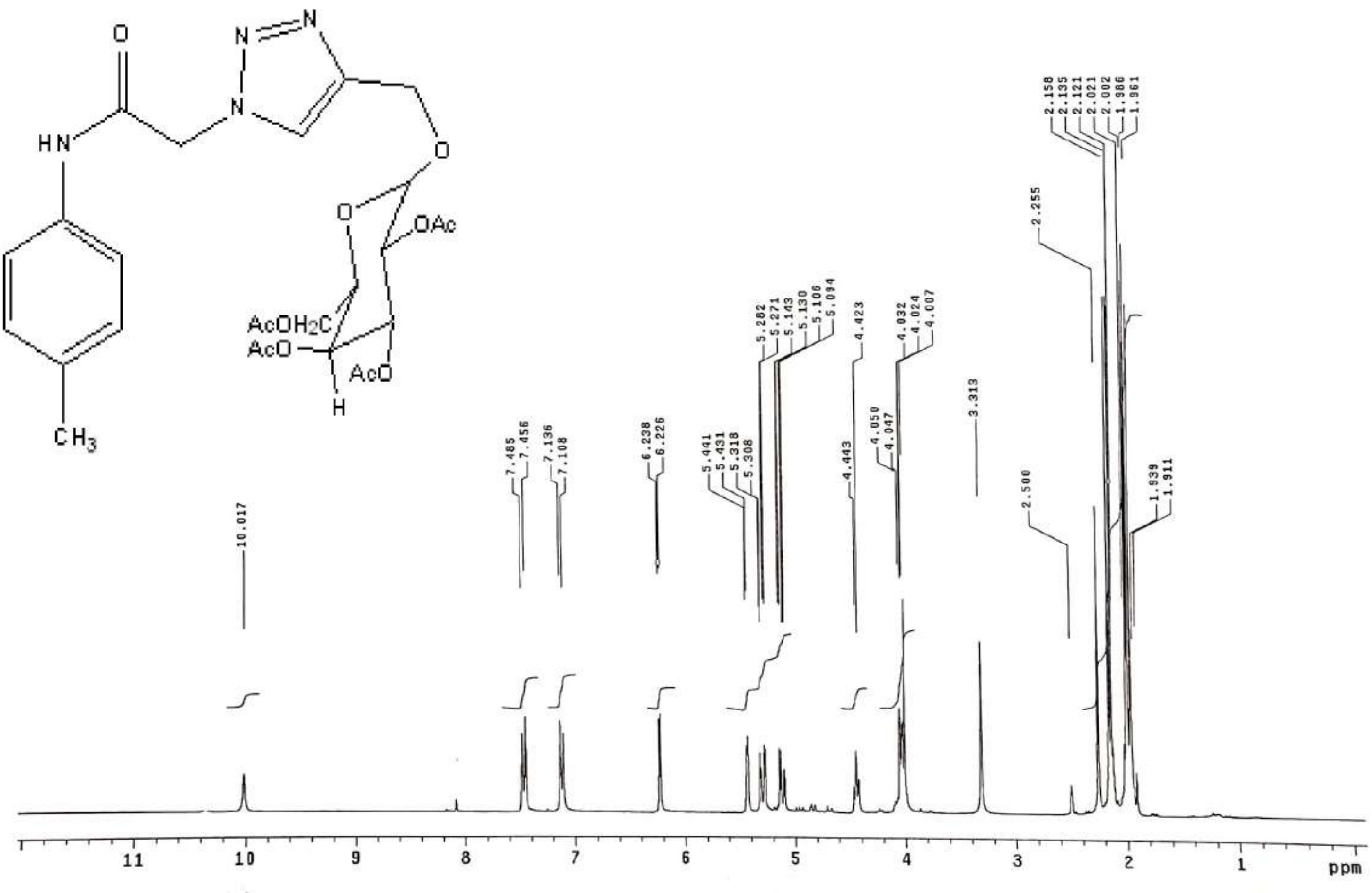

^{13}C NMR for compound 12

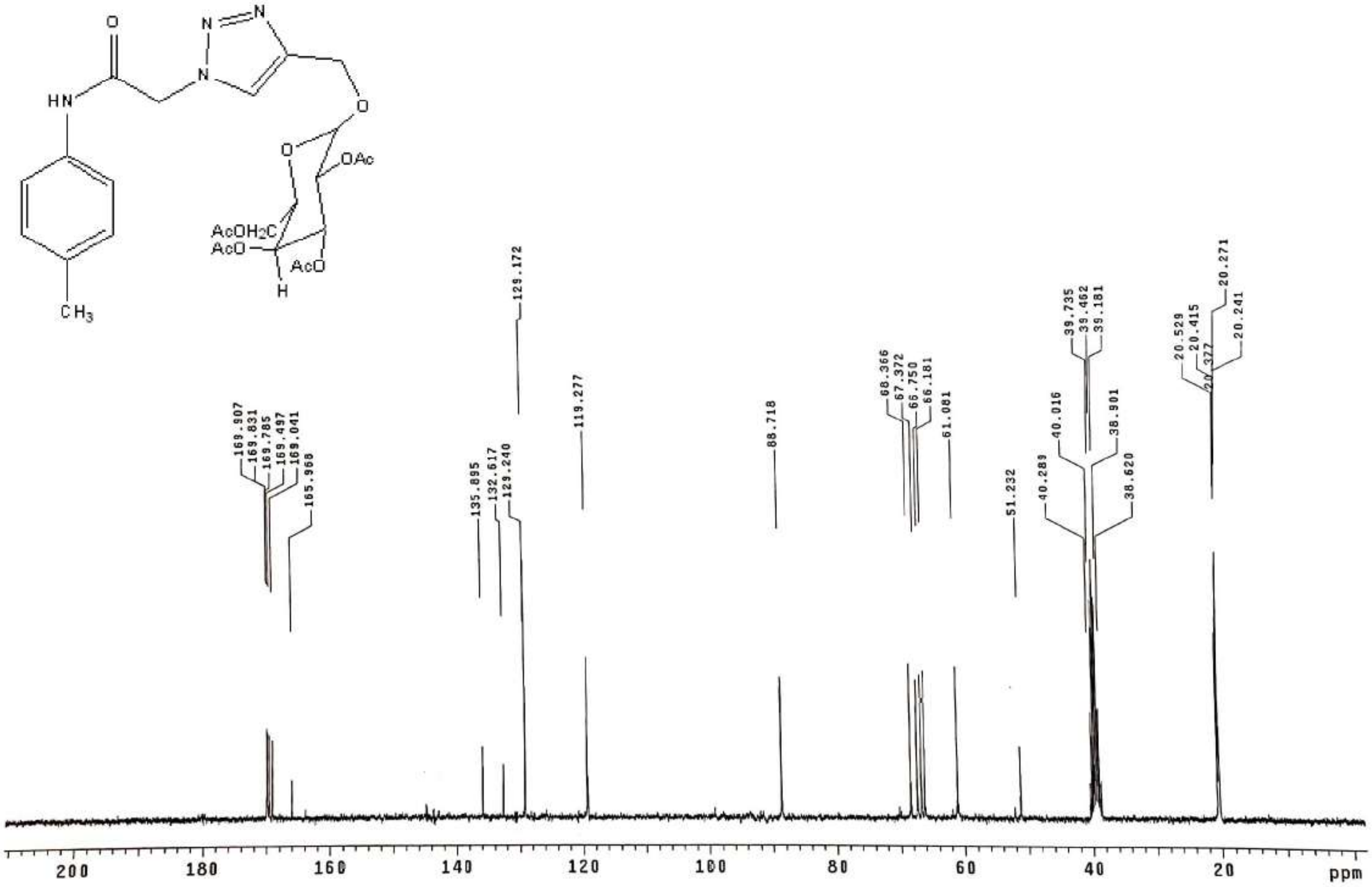

^{1}H NMR for compound 13

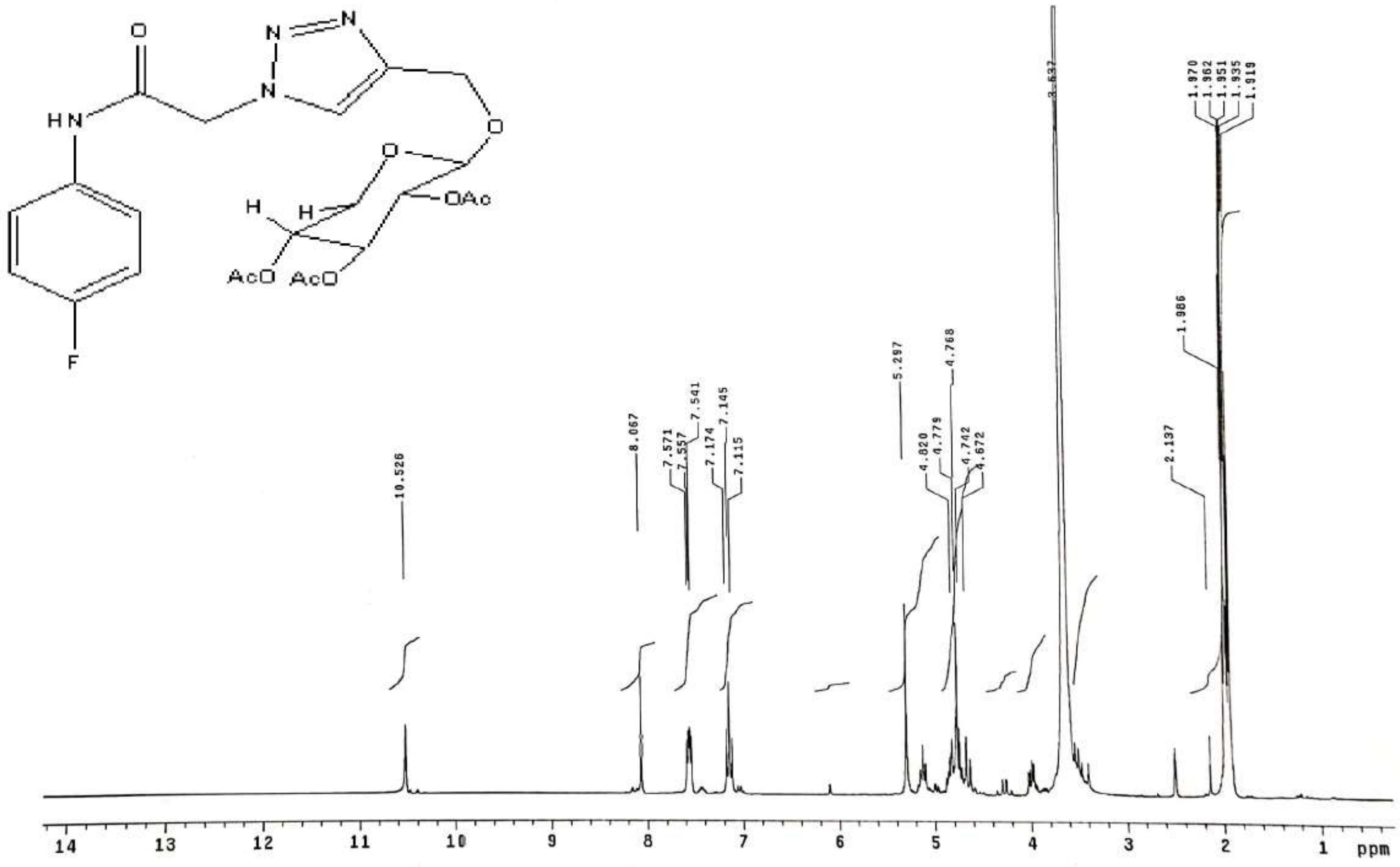

^{13}C NMR for compound 13

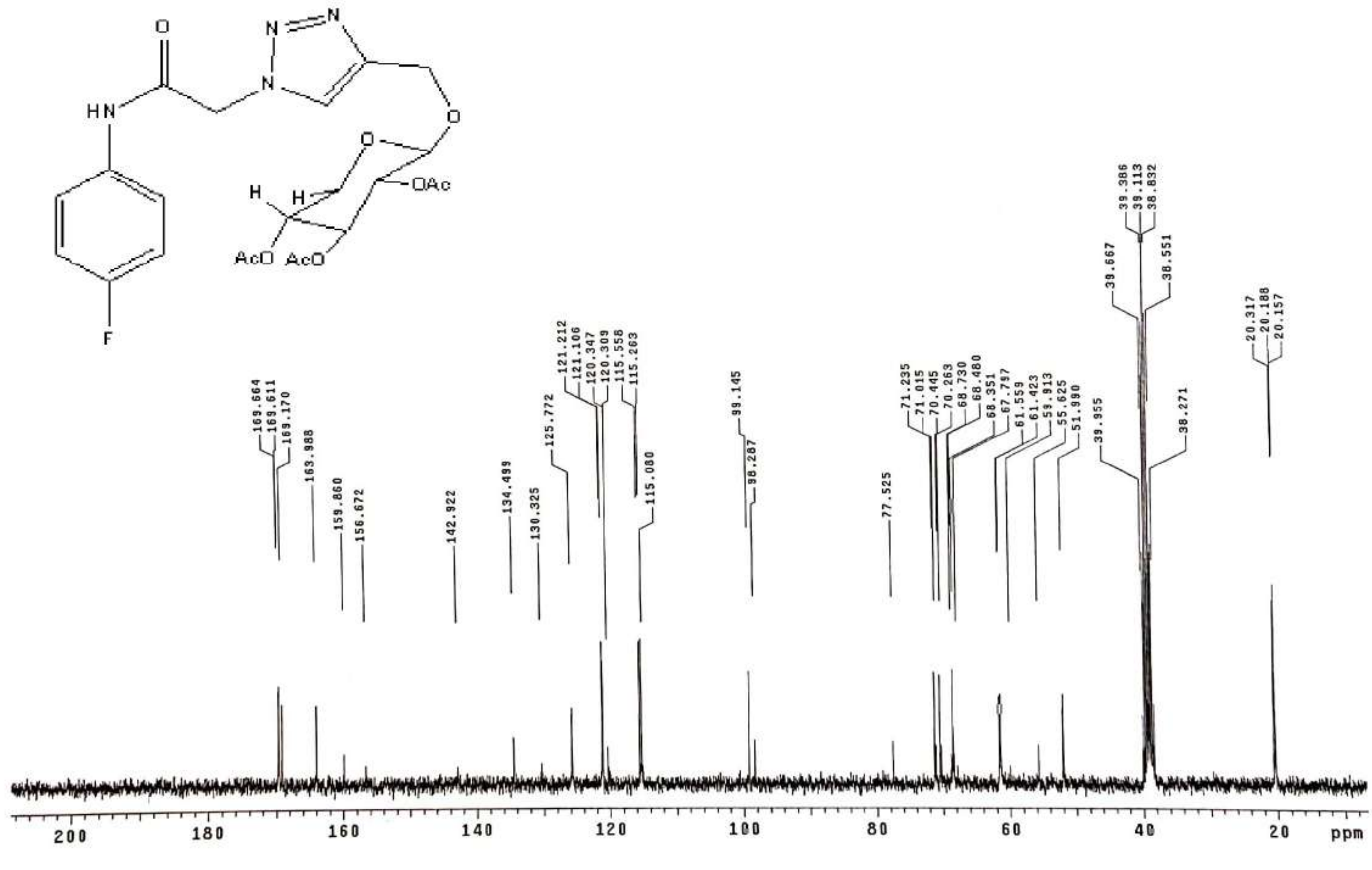

^{1}H NMR for compound 14

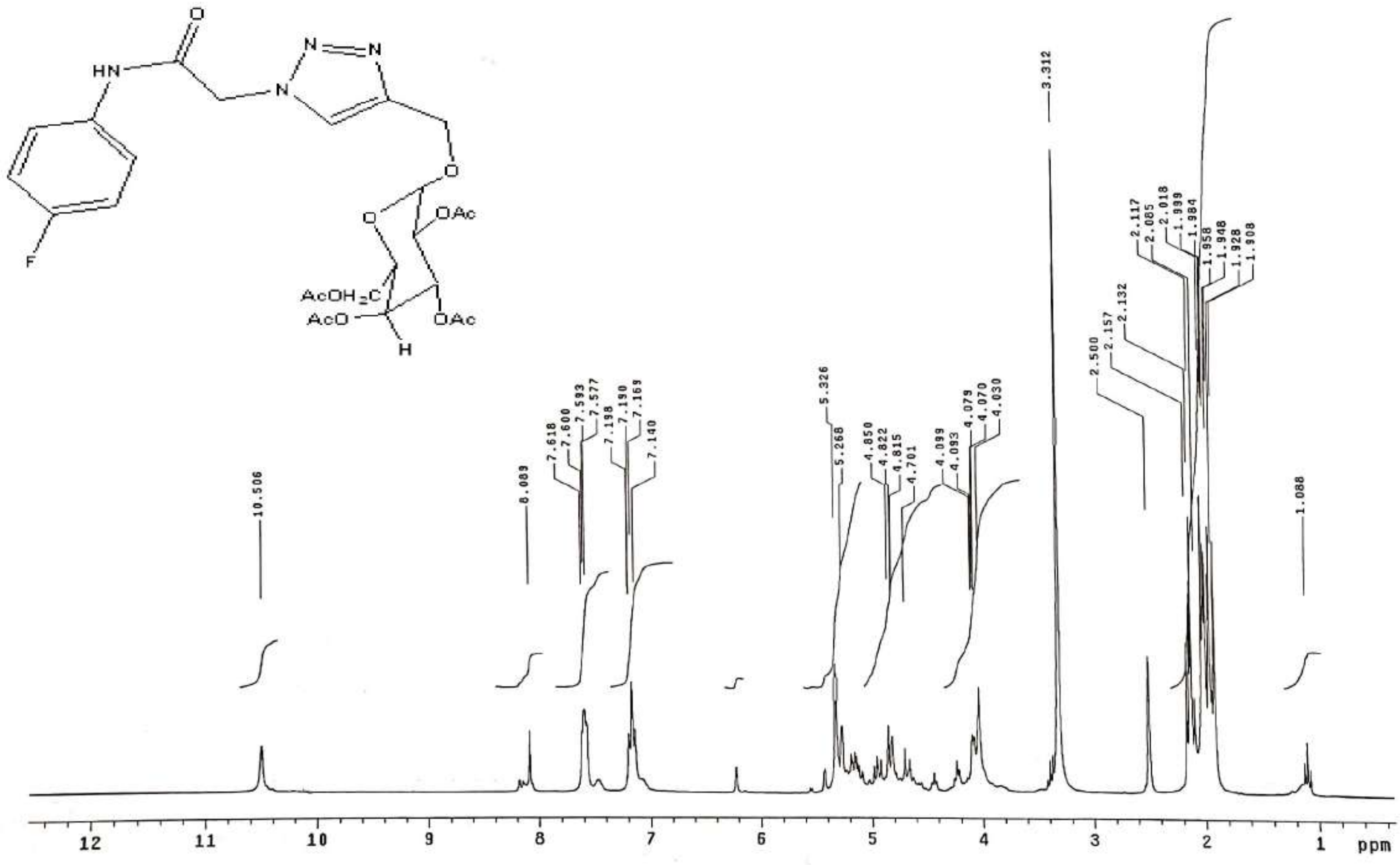

Supplement: Supplementary file 1 [file molecules-27-06960-s001.zip › molecules-1942078-supplementary.pdf]
